# Sex Dimorphism of Nonalcoholic Fatty Liver Disease (NAFLD) in *Pparg*-Null Mice

**DOI:** 10.3390/ijms22189969

**Published:** 2021-09-15

**Authors:** Mariano Schiffrin, Carine Winkler, Laure Quignodon, Aurélien Naldi, Martin Trötzmüller, Harald Köfeler, Hugues Henry, Paolo Parini, Béatrice Desvergne, Federica Gilardi

**Affiliations:** 1 Center of Integrative Genomics, Genopode, Lausanne Faculty of Biology and Medicine, CH-1015 Lausanne, Switzerland; mariano.schiffrin@gmail.com (M.S.); carine.winkler@unil.ch (C.W.); laurelausanne@gmail.com (L.Q.); aurelien.naldi@gmail.com (A.N.); Beatrice.Desvergne@unil.ch (B.D.); 2 Core Facility Mass Spectrometry, Medical University of Graz, 8036 Graz, Austria; martin.troetzmueller@medunigraz.at (M.T.); harald.koefeler@klinikum-graz.at (H.K.); 3 Centre Hospitalier Universitaire Vaudois (CHUV), Lausanne Faculty of Biology and Medicine, CH-1011 Lausanne, Switzerland; Hugues.Henry@chuv.ch; 4 CardioMetabolic Unit, Department of Medicine and Department of Laboratory Medicine, Karolinska Insititutet and Theme Inflammation and Ageing Karolinska University Hospital Huddinge, 14186 Stockholm, Sweden; paolo.parini@ki.se; 5 Faculty Unit of Toxicology, University Center of Legal Medicine, Faculty of Biology and Medicine, Lausanne University Hospital, CH-1000 Lausanne, Switzerland

**Keywords:** nonalcoholic fatty liver disease (NAFLD), sex dimorphism, lipidomics, hepatic sex-biased gene expression

## Abstract

Men with nonalcoholic fatty liver disease (NAFLD) are more exposed to nonalcoholic steatohepatitis (NASH) and liver fibrosis than women. However, the underlying molecular mechanisms of NALFD sex dimorphism are unclear. We combined gene expression, histological and lipidomic analyses to systematically compare male and female liver steatosis. We characterized hepatosteatosis in three independent mouse models of NAFLD, *ob/ob* and lipodystrophic fat-specific (*PpargF*^Δ/Δ^) and whole-body PPARγ-null (*Pparg*^Δ/Δ^) mice. We identified a clear sex dimorphism occurring only in *Pparg*^Δ/Δ^ mice, with females showing macro- and microvesicular hepatosteatosis throughout their entire life, while males had fewer lipid droplets starting from 20 weeks. This sex dimorphism in hepatosteatosis was lost in gonadectomized *Pparg*^Δ/Δ^ mice. Lipidomics revealed hepatic accumulation of short and highly saturated TGs in females, while TGs were enriched in long and unsaturated hydrocarbon chains in males. Strikingly, sex-biased genes were particularly perturbed in both sexes, affecting lipid metabolism, drug metabolism, inflammatory and cellular stress response pathways. Most importantly, we found that the expression of key sex-biased genes was severely affected in all the NAFLD models we tested. Thus, hepatosteatosis strongly affects hepatic sex-biased gene expression. With NAFLD increasing in prevalence, this emphasizes the urgent need to specifically address the consequences of this deregulation in humans.

## 1. Introduction

Nonalcoholic fatty liver disease (NAFLD) is considered as the hepatic manifestation of the metabolic syndrome and is associated with obesity, insulin resistance and diabetes. Therefore, its clinical prevalence has grown in recent years, due to the obesity epidemic. NAFLD is characterized by an excessive accumulation of triglycerides (TGs) and cholesterol esters in hepatocytes, also referred to as hepatosteatosis. The simple accumulation of fat is *per se* harmless, and the incidence of NAFLD in the adult population in Western countries is estimated to be around 25%. However, approximately 20% of patients with NAFLD develop liver inflammation, which is the hallmark of nonalcoholic steatohepatitis (NASH) [1,2]. Once developed, NASH can progress towards fibrosis and ultimately cirrhosis, which is an important risk factor for hepatocellular carcinoma [3].

One factor that seems to play a role in NASH incidence is gender, although epidemiological studies on this topic are still scarce and limited, and the available information contradictory. Whereas earlier observations suggested that NAFLD/NASH was a female-predominant condition, recent data suggest a higher prevalence in men [3]. In addition, hepatocellular carcinoma, which can be triggered by advanced liver fibrosis, is clearly sexually dimorphic in both rodents and humans, with a significantly higher incidence in males [4,5]. Finally, several reports suggest that the sex-specific fat distribution, which favors subcutaneous versus visceral depots in women, could be one of the factors contributing to the lower global metabolic risk observed in women [6,7].

However, it must be considered that so far male subjects have been favored in human and animal biomedical research, whereas women or nonhuman females have been under-represented [8]. Thus, the current observations may mostly reflect a lack of knowledge of the sex dimorphism of NAFLD. The liver is a highly sexually dimorphic organ in the situation of normal health, with hundreds of genes being differentially expressed between the two sexes [9,10]. It is thus expected that not only the development but also the consequences of NAFLD might be sex-dimorphic.

In this study, peroxisome proliferator-activated receptor gamma null mice (*Pparg*^Δ/Δ^) were used as a new model of NAFLD. PPARγ is a nuclear receptor required for adipocyte differentiation and maturation [11]. *Pparg*^Δ/Δ^ mice were obtained as described by Nadra et al. (2010) [12]. As expected from the critical role of PPARγ in adipogenesis, *Pparg*^Δ/Δ^ mice are totally deprived of adipose tissue [13] and spontaneously develop hepatosteatosis. Herein we show that, in this mouse model, hepatosteatosis evolves differently in males and females. Using a combination of transcriptomics, lipidomics and further in vivo experiments, we systematically characterize the liver phenotype of *Pparg*^Δ/Δ^ mice in both sexes in order to gain insight into the molecular mechanisms underlying sex dimorphism in NAFLD. We also pay particular attention to the expression of sex-biased genes in *Pparg*^Δ/Δ^ mice as well as distinct mouse models of hepatosteatosis, revealing an important perturbation of the sex dimorphism pattern of the liver upon steatosis.

## 2. Results

### 2.1. PPARγ-Null Mice Represent a New Model of NAFLD Exhibiting Sex Dimorphism

**Figure 1:**
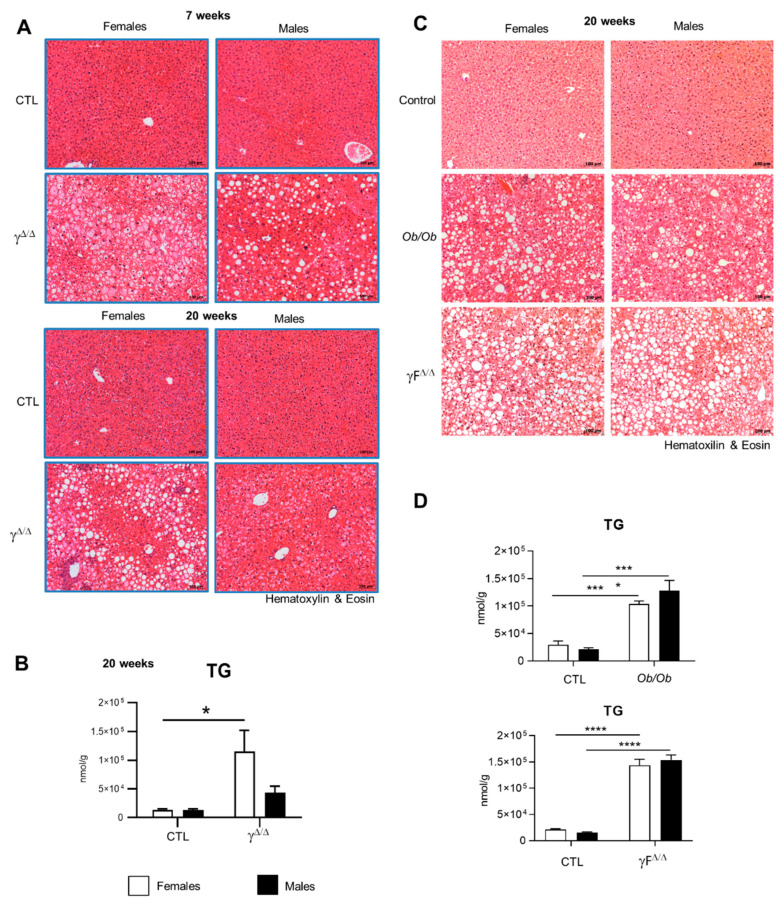
*Pparg*^Δ/Δ^ mice are a new model of NAFLD exhibiting sex dimorphism. (**A**) Hematoxylin and eosin staining of liver sections of *Pparg*^Δ/Δ^ mice and their control littermates at 7 weeks (up panels) and at 20 weeks (bottom panels). (**B**) Total hepatic TG measured in *Pparg*^Δ/Δ^ mice and their control littermates at 20 weeks. n = 3–6. (**C**) Hematoxylin and eosin staining of liver sections of *ob/ob* mice, *Adipoq-Cre^tg/+^;Pparg^fl/fl^* (*PpargF*^Δ/Δ^) mice and control mice at 20 weeks. (**D**) Total hepatic TG measured in *ob/ob* mice, *PpargF*^Δ/Δ^) at 20 weeks. n = 3–5. For (**A**,**C**), black bar corresponds to 100 µm. In (**B**,**D**), white bars are female and black bars are male data. All data were statistically treated by two-way ANOVA and Bonferroni multiple comparisons. *p* values: * <0.05, *** <0.001 and **** <0.0001.

PPARγ-null mice, hereafter called *Pparg*^Δ/Δ^ mice, are totally deprived of adipose tissue [14]. Due to the impossibility of storing lipids in adipose tissue, lipodystrophy typically triggers fat accumulation in the liver [15]. Accordingly, both male and female *Pparg*^Δ/Δ^ mice showed a massive enlargement of the liver and developed hepatic steatosis, as demonstrated by the presence of numerous lipid droplets at 7 weeks. In contrast, at 20 weeks, the liver of *Pparg*^Δ/Δ^ males exhibited fewer lipid droplets and lower triglyceride (TG) accumulation compared to *Pparg*^Δ/Δ^ females (Figure 1A,B). FFAs increased similarly in both *Pparg*^Δ/Δ^ males and females, while neither total hepatic cholesterol nor cholesterol esters were increased in *Pparg*^Δ/Δ^ mice (Supplementary Figure S1). We compared this model with two other models of NAFLD: the obese and diabetic *ob/ob* mice and the lipodystrophic *Adipoq-Cre*^tg/+^;*Pparg^fl/fl^* mice (hereafter called *Pparg*F^Δ/Δ^), in which *Pparg* is deleted in preadipocytes but is present in the rest of the body [16]. As expected, hematoxylin and eosin staining showed that both sexes had a high number of hepatic lipid droplets in these two other mouse models (Figure 1C), but with no apparent dimorphism. Consistently, neither *ob/ob* mice nor *Pparg*F^Δ/Δ^ mice showed sex dimorphism in hepatic TG levels (Figure 1D). Thus, the sex dimorphism of hepatosteatosis with higher TG storage in female vs. male is specific to the *Pparg*^Δ/Δ^ mice.

To gain insights into the development of this sex-related phenotype, we fully characterized the lipid species accumulating in the livers of male and female *Pparg*^Δ/Δ^ mice at 7 weeks, when hepatic lipid content is similar in males and females, and at 20 weeks, when the hepatic lipid content shows sexual dimorphism.

**Figure 2:**
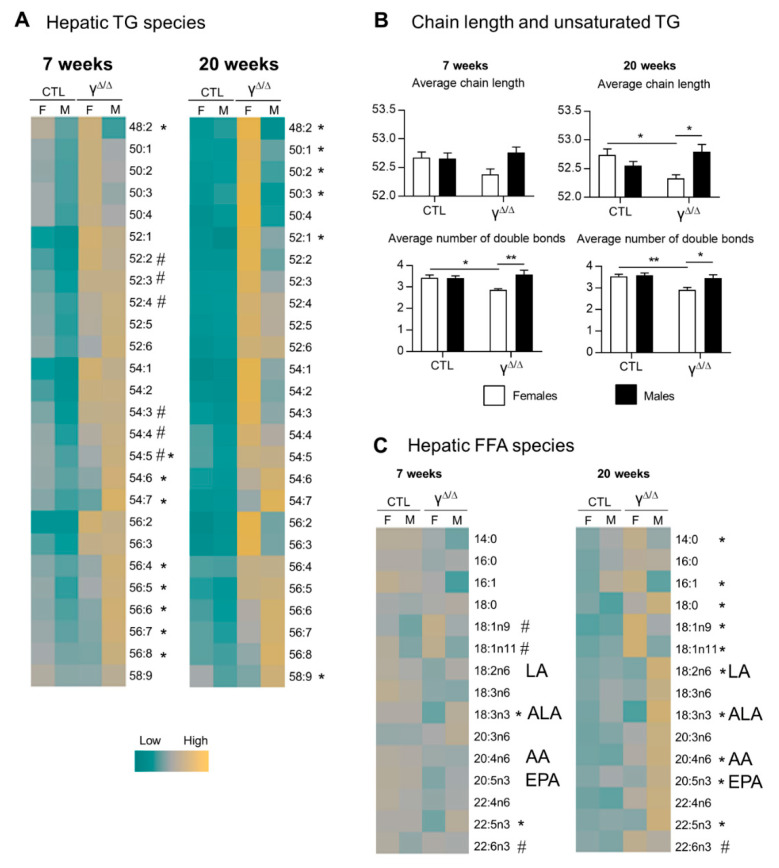
Hepatic triglyceride and FFA species in *Pparg*^Δ/Δ^ mice. (**A**) Heat map showing the different TG species. For each line, corresponding to one lipid species, the absolute values are centered to 1, and relative changes between each group are expressed in log2 (log(V/mean;2)). # and * indicate statistically significant difference (*p* < 0.05) between females and males in control and *Pparg*^Δ/Δ^ mice, respectively (Student’s *t*-test). (**B**) TG chain length and TG unsaturated average at 20 weeks, n = 5. *p* values (* <0.05, ** <0.01) were calculated by two-way ANOVA and Bonferroni multiple comparisons. (**C**) Heat map showing the different FFA species. LA, linoleic acid (18:2n6); ALA, α-linolenic acid (18:3n3); AA, arachidonic acid (20:4n6); EPA, eicosapentaenoic acid (20:5n3); n = 5. # and * indicate statistically significant difference (*p* < 0.05) between females and males in control and *Pparg*^Δ/Δ^ mice, respectively (Student’s *t*-test).

In control mice at 7 weeks, female livers had an overall slightly higher content of each TG species compared to male livers. At 20 weeks, most of these differences disappeared (Figure 2A and Supplementary Figure S2A,B). The same analyses in *Pparg*^Δ/Δ^ mice at 7 weeks revealed a remarkable pattern with a higher amount of polyunsaturated long-chain TGs in *Pparg*^Δ/Δ^ males but a higher amount of short-chain and more saturated TGs in *Pparg*^Δ/Δ^ females, whereas no sex dimorphism was observed in that respect in control mice. The same phenotype was accentuated at 20 weeks, while *Pparg*^Δ/Δ^ females had higher total TG content compared to males, suggesting that this pattern is independent of total hepatic TG content (Figure 2A,B).

The profile of hepatic FFAs, from which TGs are synthesized, showed only few differences at 7 weeks between *Pparg*^Δ/Δ^ males and females. In contrast, the FFA profile at 20 weeks reproduced the same pattern found in TGs (Figure 2C and Supplementary Figure S2C).

Similar analyses were performed in 20 weeks *ob/ob* mice. Unlike in *Pparg*^Δ/Δ^ mice, short-hydrocarbon-chain TGs were more concentrated in *ob/ob* males compared to females, while FFA species did not show sex dimorphism (data not shown).

In summary, *Pparg*^Δ/Δ^ males and females showed sexual dimorphism in the hepatic content of TG and FFA species. In addition, females exhibit more short and/or saturated hydrocarbon chain TGs and FFAs whereas males have more long and/or polyunsaturated TGs and FFAs.

### 2.2. Distribution Pattern of Sex-Biased Genes in the Liver of CTL and Pparg^Δ/Δ^ Mice

To define the signature of the steatotic liver in male and female *Pparg*^Δ/Δ^ mice and the genes/mechanisms underlying the observed sex dimorphism of NAFLD, microarray analyses were performed at 20 weeks.

**Figure 3:**
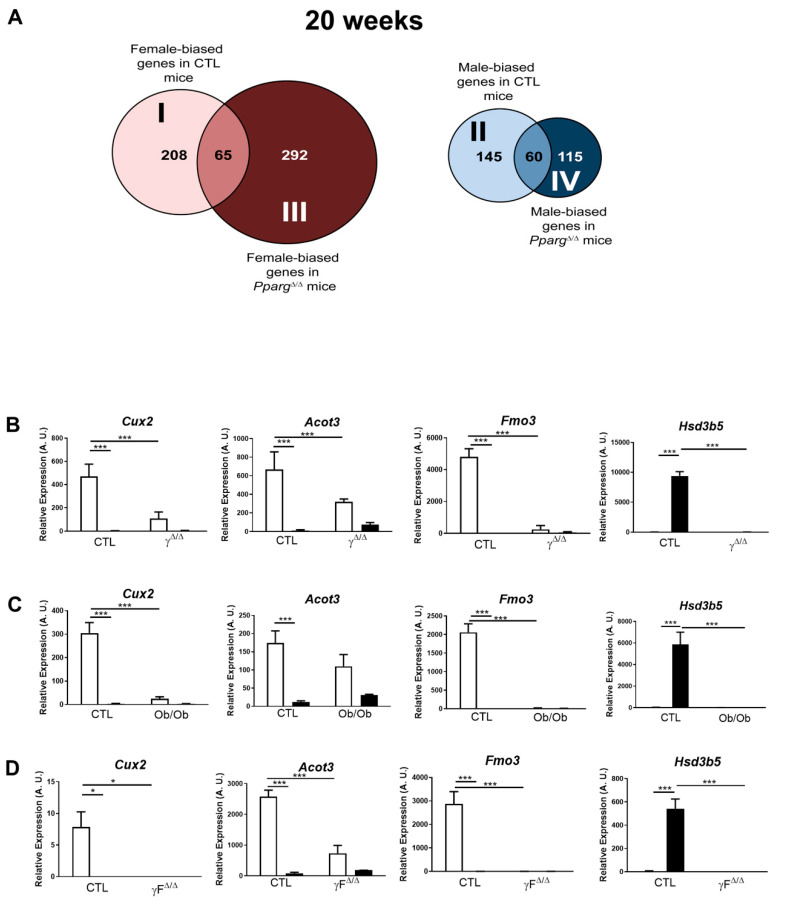
Perturbation of hepatic sex-biased gene expression by NAFLD. (**A**) Distribution of hepatic sex-biased genes measured by microarray analysis in *Pparg*^Δ/Δ^ mice at 20 weeks. Global computation of *p*-value adjustment was performed for the two comparisons, with adjusted *p* value < 0.05 and no cutoff with fold change. “I” represents the group of genes that are female-biased only in CTL mice. “II” represents the group of genes that are male-biased only in CTL mice. “III” represents the group of genes that are not sex-biased in CTL but become female-biased in *Pparg*^Δ/Δ^ mice. “IV” represents the group of genes that are not sex-biased in CTL but become male-biased in *Pparg*^Δ/Δ^ mice. (**B**) Hepatic expression profile of *Cux2, Acot3, Fmo3* and *Hsd3b5* was confirmed by RT-qPCR in *Pparg*^Δ/Δ^ mice at 20 weeks (n = 4–7) and was measured in (**C**) *Ob/Ob* mice (n = 5) and in (**D**) *Adipoq-Cre^tg/+^;Pparg^fl/fl^* (*PpargF^Δ/Δ^*) (n = 3). White bars are female and black bars are male data. *p* values (* <0.05, *** <0.001) were calculated by two-way ANOVA and Bonferroni multiple comparisons.

The hepatic expression of many genes is physiologically different between males and females. The genes more expressed in females compared to males and the opposite are referred to as “female-biased” or “male-biased” genes, respectively. Disruption of this natural dimorphism may lead to physiopathological disorders. We thus compared the sets of female-biased and male-biased genes in CTL and *Pparg*^Δ/Δ^ mice. An intriguing pattern emerged, as most of the hepatic sex-biased genes in CTL mice lose their sex dimorphism in *Pparg*^Δ/Δ^ mice (e.g., less than one-third of female-biased genes in CTL mice remain female-biased in *Pparg*^Δ/Δ^ mice). Reciprocally, an important number of non-sex-dimorphic genes in CTL become sex-biased in *Pparg*^Δ/Δ^ mice (Figure 3A).

In order to find the main biological pathways impacted by this particular pattern of sex-biased genes in *Pparg*^Δ/Δ^ mice at 20 weeks, we performed gene ontology (GO) analysis, using the bioinformatics tool DAVID GO (https://david.ncifcrf.gov/summary.jsp, analyses performed from September 2015 to September 2016). We divided the overall set of sex-biased genes into four subsets, as shown in Figure 3A. Table 1 lists the main GO terms represented in each subset. Further analyses also take into account the specific genes that GO groups in these categories, as listed in Supplementary Table S2.

### 2.3. Perturbation of the Physiological Sex-Biased Gene Expression by NAFLD

Subsets I and II include genes that are physiologically gender-biased in WT but lose their sex dimorphism in *Pparg*^Δ/Δ^ mice. In particular, *subset I corresponds* to female-biased genes in WT but not in *Pparg*^Δ/Δ^ mice. In this group, we found *Cux2*, which is a highly female-specific liver transcription factor, involved in male-biased gene repression and female-biased gene induction [17]. *Cux2* expression was reduced by more than 65% in *Pparg*^Δ/Δ^ females compared to control females at 20 weeks (Figure 3B). In addition, there is a major representation of genes involved in drug metabolism such as the cytochrome P450 family including *Cyp4a10*, the flavin-containing monooxygenases (*Fmo1*, *2*, *3* and *4*) and the glutathione S transferase. The same genes are also found under the GO terms arachidonic acid and linoleic acid metabolism. This strong sex dimorphism in drug metabolism-related genes in the healthy liver is known (reviewed by DJ Waxman and MG Holloway [9]), while its disruption in *Pparg*^Δ/Δ^ mice raises questions about possible alteration of the normal drug metabolism.

*Subset II corresponds* to male-biased genes in CTL but not in *Pparg*^Δ/Δ^ mice. One major observation within this subset concerns the steroid dehydrogenase activity that includes genes of the *Hsd3b* family (*Hsd3b2*, *Hsd3b4*, *Hsd3b5*). These genes are important for the biosynthesis of active steroid hormones. In particular, *Hsd3b5* is highly expressed in the liver in a male-specific manner [18] and is dramatically downregulated in *Pparg*^Δ/Δ^ males, reaching the very low levels observed in females (Figure 3B).

Interestingly, the sex-biased expression profile of a panel of these sex-biased genes, including *Cux2*, *Acot3*, *Fmo3* and *Hsd3b5*, was similarly dampened in the two other models of NAFLD previously used in this study, namely the *ob/ob* and *PpargF*^Δ/Δ^ mice (Figure 3C,D). These results seem against a possible involvement of these gene sets in the development of the sex-dimorphic lipid accumulation observed in *Pparg*^Δ/Δ^ mice, while they suggest that NAFLD, rather than PPARγ, has an impact on the physiological hepatic gender dimorphism of gene expression. Given the involvement of these genes in drug metabolism, our observations raise questions about the possible consequences of NAFLD on pharmacological responses, which would deserve further studies.

### 2.4. Modulation of Pathways Involved in Lipid Droplet Formation, Storage and Secretion in Pparg^Δ/Δ^ Mice

The two remaining subsets highlighted by microarray analysis (III and IV) include genes that are not physiologically sex-biased but acquired a sex-dimorphic gene expression in *Pparg*^Δ/Δ^ mice.

*Subset III corresponds* to female-biased genes in *Pparg*^Δ/Δ^ but not in CTL mice. Three main domains, immune response, cell activation and lipid metabolism, are associated with this subset, which is principally composed of genes involved in immune responses and cell activation and genes involved in mono- and polyunsaturated fatty acids (*Scd2, Fads1* and *Fads2*), as well as including genes involved in arachidonic acid metabolism (*Tbxas1* and *Hgpds*). This subset is likely to play an important role in the sex dimorphism of the fatty liver in *Pparg*^Δ/Δ^ mice.

*Subset IV corresponds* to male-biased genes in *Pparg*^Δ/Δ^ but not in CTL mice. The GO categories in this subset regroup cellular damages at the level of membranes, but also at the DNA level, converging towards the P53 pathway, as represented by *Aen* (apoptosis-enhancing nuclease) and *Jmy* (junction-mediating and regulatory protein) genes. This subset also includes genes involved in oxidation and mitochondrial functions (Supplementary Table S2).

**Figure 4:**
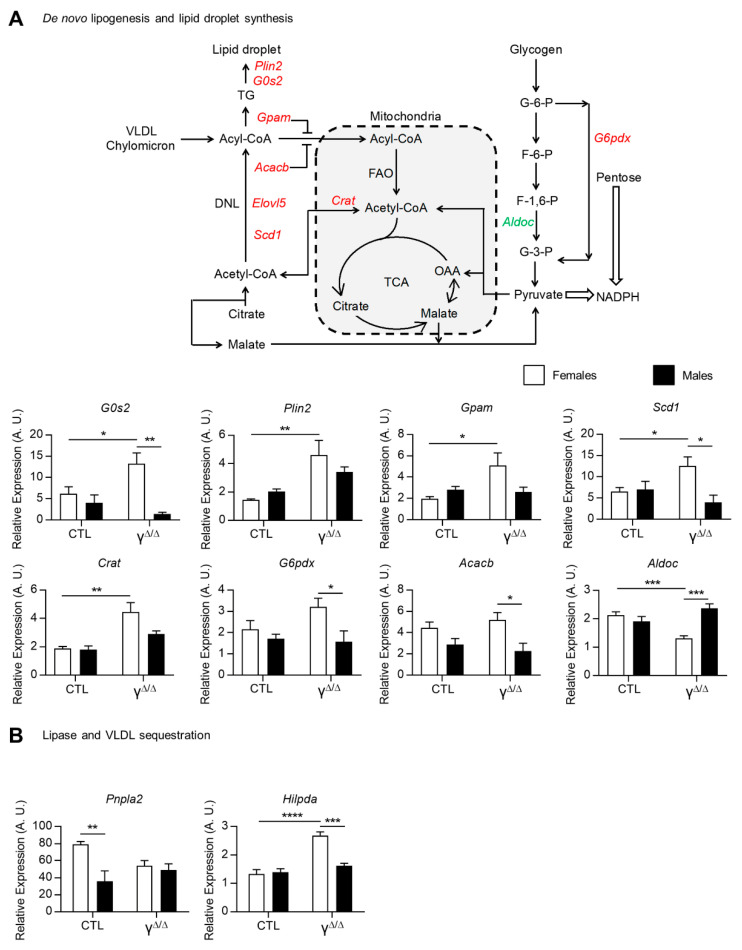
Pathways involved in lipid droplet formation, storage and secretion. (**A**) Representative scheme of the biochemical pathways of de novo lipogenesis and lipid droplet synthesis: upregulated and downregulated genes in *Pparg*^Δ/Δ^ females, but not in *Pparg*^Δ/Δ^ males, are in red and green, respectively. DNL, de novo lipogenesis; TCA, citric acid cycle; OAA, oxaloacetate; VLDL, very-low-density lipoprotein; G-6-P, glucose 6-phosphate; F-6-P, fructose 6-phosphate; F-1,6-P, fructose 1,6-bisphosphate; G-3-P, glyceraldehyde 3-phosphate. Gene expression of perilipin 2 (*Plin2*); G0/G1 switch gene 2 (*G0s2*); mitochondrial glycerol-3-phosphate acyltransferase (*Gpam*); acetyl-CoA carboxylase beta (*Acacb*); stearoyl-Coenzyme A desaturase 1 (*Scd1*); ELOVL family member 5, elongation of long-chain fatty acids (*Elovl5*); carnitine acetyltransferase (*Crat*); the glucose-6-phosphate dehydrogenase X-linked (*G6pdx*); and aldolase C, fructose-bisphosphate (*Aldoc*). (**B**) Adipose triglyceride lipase (ATGL or *Pnpla2*) and hypoxia-inducible lipid droplet-associated (*Hilpda*) gene expression measured by RT-qPCR at 20 weeks. n = 3–9. White bars are female and black bars are male data. *p* values (* <0.05, ** <0.01, *** <0.001 and **** <0.0001) were calculated by two-way ANOVA and Bonferroni multiple comparisons.

Given the higher steatosis development in *Pparg*^Δ/Δ^ female mice, we further explored the genes and/or pathways particularly highlighted in subset III. More particularly, the expression of genes involved in de novo lipogenesis and in lipid droplet formation, such as *G0s2, Plin2, Gpam, Scd1, Crat, G6pdx, Acacb* and *Elovl5*, were all upregulated only in *Pparg*^Δ/Δ^ females and/or became female-biased in *Pparg*^Δ/Δ^ mice at 20 weeks. This increased expression was validated by qRT-PCR, as shown in Figure 4A. In contrast, aldolase C fructose-bisphosphate (*Aldoc*) was only downregulated in *Pparg*^Δ/Δ^ females. This could favor the use of glycogen and/or glucose to feed the pentose phosphate pathway. The adipose triglyceride lipase ATGL (*pnpla2*), which is involved in intracellular degradation of TGs, showed female-biased expression in control mice, but not in *Pparg*^Δ/Δ^ mice. Finally, the hypoxia-inducible lipid droplet-associated gene (*Hilpda*), which inhibits hepatic triglyceride secretion [19], was upregulated only in *Pparg*^Δ/Δ^ females (Figure 4B). The profile of the two latter genes is contributing, at least in part, to the higher hepatic TG content in females compared to males.

Altogether, gene expression analysis showed that dysregulations of all aspects of lipid synthesis, storage and secretion concur in determining higher hepatic TG levels in *Pparg*^Δ/Δ^ females vs. males.

### 2.5. Analyses of Lipids and Lipid Pathways Involved in Cell Signaling and Inflammation

Subset III of genes (sex-biased in female *Pparg*^Δ/Δ^ mice but not in female CTL mice) also highlighted genes involved in immune response and cell activation. Using the combination of lipidomics and transcriptomics, we thus further analyzed the lipids involved in cell signaling and inflammation. Ceramides, which are found in high concentrations in cell membranes, participate in a variety of cellular signaling pathways in differentiation, proliferation and cell death [20]. At 7 weeks, total ceramide content in the liver of control mice was significantly higher in males compared to females, a sex dimorphism that was attenuated in *Pparg*^Δ/Δ^ mice. The same pattern was seen at 20 weeks, in both control and *Pparg*^Δ/Δ^ mice, although the high variability precludes statistical differences for most species (Supplementary Figure S3A).

Among the FFA species, we more specifically analyzed omega-3 and omega-6 fatty acids because of their correlation with obesity and the progression to steatohepatitis [21]. At 20 weeks, the concentrations of omega-3 and omega-6 and the omega-6/omega-3 ratio were higher in *Pparg*^Δ/Δ^ males compared to *Pparg*^Δ/Δ^ females, suggesting that males could be more prone to progress to steatohepatitis (Supplementary Figure S3B).

**Figure 5:**
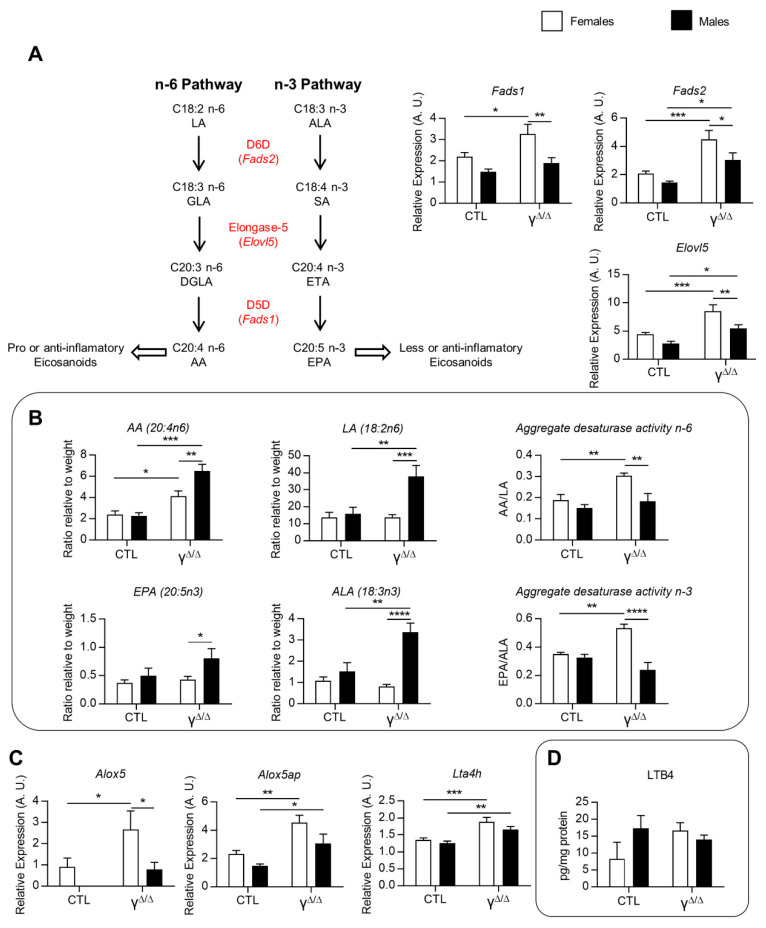
Linoleic acid (LA) and arachidonic acid (AA) metabolism and eicosanoids. (**A**) Left graphs: Pathways with main genes involved in essential FFA transformation and their hepatic gene expression. ALA, α-linolenic acid (18:3n3); SA, stearidonic acid; ETA, eicosatetraenoic acid; EPA, eicosapentaenoic acid (20:5n3); LA, linoleic acid (18:2n6); GLA, γ-linolenic acid; DGLA, dihomo-γ-linolenic acid; AA, arachidonic acid (20:4n6). D5D (Δ5-desaturase), D6D (Δ6-desaturase) and elongase-5 enzymes are encoded by *Fads1, Fads2* and *Elovl5*, respectively. Right graphs: Relative expression of the three genes corresponding to these enzymes, n = 3–9. (**B**) Aggregate desaturase activity, calculated as the AA/LA ratio and the EPA/ALA ratio, reflects the FADS1 and FADS2 activity [22], n = 3–5. (**C**) Hepatic expression of genes involved in leukotriene B4 (LTB4) synthesis from AA, n = 9. ND: not detectable. *Alox5* was almost not detectable by RT-qPCR. 5-Lipoxygenase-associated protein (*Alox5ap*) and leukotriene A4 hydrolase (*Lta4h*). (**D**) Hepatic LTB4, n = 5–9. White bars are female and black bars are male data. *p* values (* <0.05, ** <0.01, *** <0.001 and **** <0.0001) were calculated by two-way ANOVA and Bonferroni multiple comparisons.

Eicosanoids represent a third class of lipids with important roles in inflammation. As we indeed see in Figure 2C, the hepatic levels of two essential FFAs, linoleic acid (LA, 18:2n-6) and α-linolenic acid (ALA, 18:3n3), are lower in *Pparg*^Δ/Δ^ females compared to *Pparg*^Δ/Δ^ males. LA, the major vegetal dietary n-6 PUFA and precursor of arachidonic acid (AA), is considered as a proinflammatory compound, whereas ALA can be metabolized into anti-inflammatory molecules such as eicosapentaenoic acid (EPA, 20:5n3). We thus explored the expression of genes involved in LA and ALA metabolism. mRNA levels of delta-5-desaturase (*Fads1*), delta-6-desaturase (*Fads2*) and elongase-5 (*Elovl5*), which are involved in AA and EPA formation from LA and ALA, respectively, were upregulated only in *Pparg*^Δ/Δ^ females (Figure 5A). Accordingly, the ratios AA/LA and EPA/ALA, which reflect the total activity of these enzymes [22], were higher in *Pparg*^Δ/Δ^ females compared to *Pparg*^Δ/Δ^ males (Figure 5B). Nonetheless, EPA and AA, which are the final products of these enzymes, showed a lower hepatic content in *Pparg*^Δ/Δ^ females compared to *Pparg*^Δ/Δ^ males (Figure 5B). In parallel, we found that 5-lipoxygenase (*Alox5*), 5-lipoxygenase activating protein (*Alox5ap*) and leukotriene-A4-hydrolase (*Lta4h*), are upregulated in *Pparg*^Δ/Δ^ females (Figure 5C), suggesting a possible increased conversion of AA into eicosanoids, known to play an important role in the onset and progression of inflammation in the liver [23,24]. We thus measured the full set of eicosanoids and other FFA derivatives in the livers of control and *Pparg*^Δ/Δ^ mice at 20 weeks. There were no significant differences in the levels of AA derivatives taken individually, as observed for leukotriene B4 (LTB4) (Figure 5D). However, the sum of AA derivatives synthesized through the Cox pathway, including prostaglandins, was higher in *Pparg*^Δ/Δ^ females compared to control females. This was mainly due to the increased levels of the most abundant hepatic prostaglandin, PGF2a. In contrast, no differences in the Lox pathway were found comparing *Pparg*^Δ/Δ^ to control males (Supplementary Figure S3C).

Altogether, ceramides and the omega-6/omega-3 fatty acid ratio suggested that males may be more prone to steatohepatitis. However, females exhibited a higher activity of the Cox pathway. This might explain at least in part the lower AA level in the liver of *Pparg*^Δ/Δ^ females compared to *Pparg*^Δ/Δ^ males, via a higher transformation of AA into eicosanoid derivatives.

Given the importance of lipids and some derivatives in the modulation of the inflammatory response, we thus explored whether the observed changes influence the progression of *Pparg*^Δ/Δ^ hepatosteatosis to the more severe states. As shown in Supplementary Figure S4, plasmatic levels of aspartate (ASAT) and alanine aminotransferases (ALAT), both markers of liver damage, as well as the expression levels of proinflammatory genes, were increased, although modestly. *Elane* and osteopontin (*Spp1*), which are linked to neutrophil infiltration, were more particularly increased.

In humans, a major complication of nonalcoholic steatohepatitis (NASH), following NAFLD, is liver fibrosis. We thus challenged the mice with a profibrotic diet for 6 weeks. In control mice, the diet induced hepatosteatosis and collagen deposition in both sexes and an upregulation of fibrotic markers such as *Acta2, Col1a1, Mmp13* and *Timp1,* with no statistical differences between males and females (Supplementary Figure S5). In *Pparg*^Δ/Δ^ mice, collagen deposition and fibrotic markers were already increased under chow diet. The profibrotic diet triggered a further modest upregulation of fibrotic markers, but without sex dimorphism in collagen deposition. Thus, a profibrotic diet provoked further signs of moderate hepatic inflammation and fibrosis in both male and female *Pparg*^Δ/Δ^ mice, with no detectable sex dimorphism. The fibrotic phenotype induced by the profibrotic diet in *Pparg*^Δ/Δ^ mice was comparable to that obtained with the same diet in CTL mice.

### 2.6. Relationship between Hormonal Status and Sex Dimorphism of Hepatic Lipid Accumulation

**Figure 6:**
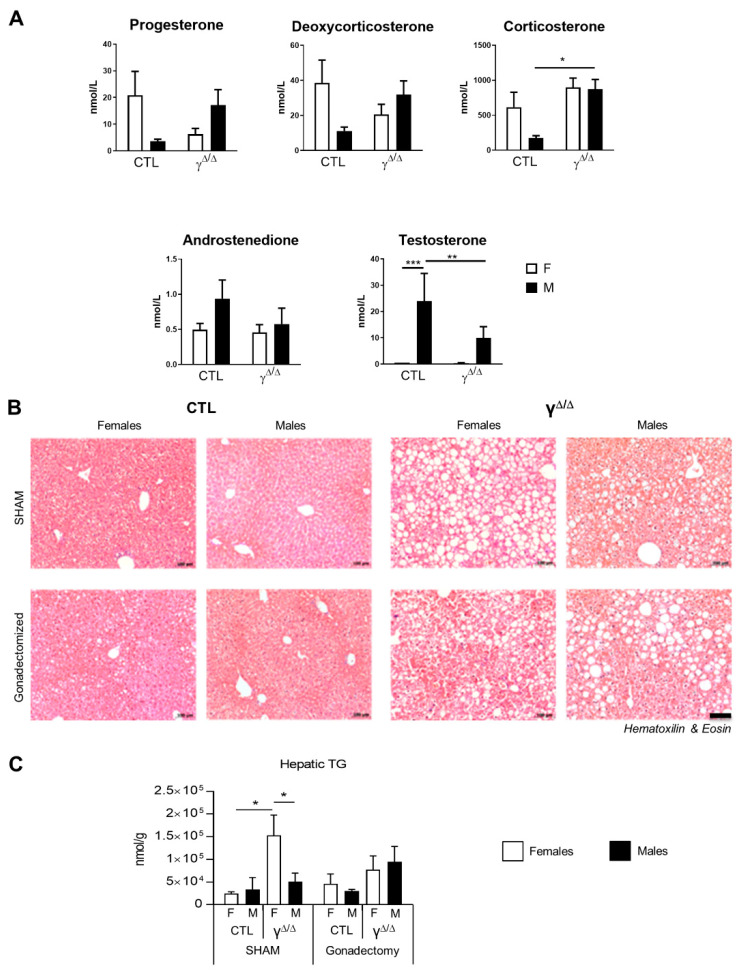
Role of sex hormones in the hepatosteatotic phenotype in *Pparg*^Δ/Δ^ mice. (**A**) Plasmatic steroid hormones measured by mass spectrometry. n = 6–9. Progesterone and deoxycorticosterone show a *p* value lower than 0.05 for the 2-way ANOVA interaction. (**B**) H&E staining of liver sections and (**C**) hepatic TG content measured in ovariectomized and castrated mice. n = 4–9. In (**B**), black bar corresponds to 100 µm. In (**A**,**C**), white bars are female and black bars are male data. *p* values (* <0.05, ** <0.01 and *** <0.001) were calculated by two-way ANOVA and Bonferroni multiple comparisons.

Sex hormones are important regulators of hepatic lipid metabolism [25]. We thus explored the hormonal status of *Pparg*^Δ/Δ^ mice by looking at the plasmatic levels of the various steroid hormones. Intriguingly, testosterone and androstenedione are significantly reduced in *Pparg*^Δ/Δ^ male mice compared to control mice, while progesterone, deoxycorticosterone and corticosterone are increased (Figure 6A), indicating some perturbation of the sex-hormone homeostasis.

To determine the role of sex steroid hormones in *Pparg*^Δ/Δ^ sex dimorphism, gonadectomy was performed between the ages of 4 and 6 weeks, prior to sexual maturation, and the resulting phenotype was analyzed at 20 weeks. The effectiveness of both castration and ovariectomy was demonstrated by the profound decrease in testosterone, progesterone and androstenedione in males and by the lack of estrogen cycle in female mice (Supplementary Figure S6). Importantly, hepatic TG content at 20 weeks was no longer dimorphic in gonadectomized mice, due to an increase in the lipid load in males and a decrease in the lipid load in females (Figure 6B,C). This confirmed the role of sex hormones in liver lipid accumulation and suggests a potential cross-talk between sex hormones and PPARγ in the onset of the sex-dependent hepatic lipid accumulation observed in *Pparg*^Δ/Δ^.

## 3. Discussion

Our study highlights a progressive sex dimorphism of NAFLD in a new lipodystrophic mouse model. NAFLD development becomes sex-dimorphic at 20 weeks, with *Pparg*^Δ/Δ^ females showing high levels of hepatic lipid droplets and triglycerides while *Pparg*^Δ/Δ^ males present limited hepatosteatosis. Such sex-dimorphic phenotype is observed only in the presence of sex hormones in *Pparg*^Δ/Δ^ mice, suggesting a cross-regulation between PPARγ and sex hormones in liver lipid metabolism. Whereas the female-biased liver steatosis was not reproduced in other mouse models of NAFLD, severe deregulation in the liver of sex-biased genes occurs in all three hepatosteatosis models that were tested.

Limitations of this study mainly stand along the fact that the liver sex dimorphism we herein characterized seems specific to one mouse model. However, it still provides some means to address the complexity of this quite uncharted biological phenomenon, and it gives even more importance to the features shared by all three NAFLD mouse models we have tested.

Lipidomics data in mouse liver addressing the differences between males and females are still quite limited. A recent report showed a transient difference in the saturation index of fatty acids in the livers of wild-type C57BL/6 males and females [26]. In our study, control females show higher abundance of several medium-chain TG species compared to males, and these differences disappeared at 20 weeks. Most interestingly, this pattern is different in *Pparg*^Δ/Δ^ mice, with higher levels of long-chain TG species in males at 7 weeks, while, at 20 weeks, the higher hepatic TG content in *Pparg*^Δ/Δ^ females mainly relies on TGs with short-and/or highly saturated hydrocarbon-chains. Variations in chain length and saturation of hepatic TGs were also observed when comparing fasting and high-fat diet (HFD) conditions in C57BL/6 male mice [27], and such differences were associated with the different energy status of these conditions. Indeed, the high rate of mitochondrial β-oxidation upon fasting could explain the reduction in fasted livers of short fatty acids, which can be directly oxidized. Along this line, the profile of hepatic TG species in *Pparg*^Δ/Δ^ males is similar to that observed in fasted mice, whereas the TG pattern of *Pparg*^Δ/Δ^ females is closer to that of HFD-fed mice. However, hepatic gene expression of markers of β-oxidation upon fasting does not reveal sex dimorphism in *Pparg*^Δ/Δ^ (data not shown). Nevertheless, *Pparg*^Δ/Δ^ female mice seem more prone to store efficiently the hepatic lipid overload compared to males, as shown by the female-specific overexpression of genes involved in lipid storage.

Sex effects were reported also for the production of fatty acid derivatives, which are of particular interest because of their ability to modulate inflammation, but studies mainly focused on the kidney and on one or two eicosanoids [28,29]. However, a recent report comprehensively characterized oxylipins in male and female rat livers and found sex effects in the abundance of 40% of them, with most of them higher in males [30]. At 20 weeks, we found a similar sex-dependent trend in control mice and an increase in the sum of AA derivatives synthesized through the Cox pathway in *Pparg*^Δ/Δ^ females, whereas no alterations were found in *Pparg*^Δ/Δ^ males.

Sex steroid hormones influence hepatic lipid metabolism through the activation of sex hormone receptors [25]. The differences between males and females in the circulating levels of sex hormones were slightly dampened in *Pparg*^Δ/Δ^ mice. Sex-hormone activity has a direct effect on hepatic lipid accumulation. Estrogens decrease liver cholesterol and triglyceride concentrations only in females [31], while tamoxifen, a potent estrogen receptor antagonist, causes severe steatosis progressing towards NASH [32]. Along this line, male but not female mice with aromatase gene deletion develop hepatic steatosis that can be rescued by estrogen treatment [33]. On the one hand, these reports suggest that estrogen receptor signaling is negatively correlated with level of hepatosteatosis in both sexes. On the other hand, androgen receptor (AR) signaling seems protective against hepatosteatosis in a sex-dimorphic manner. Male but not female mice lacking AR in the liver develop hepatosteatosis and insulin resistance upon HFD [34]. Interestingly, in *Pparg*^Δ/Δ^ mice, the impairment of sex-hormone activity through gonadectomy does not worsen hepatosteatosis in females, while it increases hepatic lipid droplet accumulation in males and suppresses sex-related differences in hepatosteatosis. These observations suggest that the hepatic phenotype of *Pparg*^Δ/Δ^ mice depends on the sex-hormone activity and highlight a cross-regulation between PPARγ and sex hormones.

An important feature of the liver is the strong sex dimorphism affecting gene expression [10]. The growth hormone (GH) secretory patterns, highly pulsatile in males and more continuous in females, determine the hepatic sex-biased expression of a high number of genes [35]. Interestingly, patients with NAFLD have low GH production and/or hepatic GH resistance [36,37]. In mice, GH inhibits de novo lipogenesis through inhibition of glycolysis [38,39]. The signal transducer and activator of transcription 5b (STAT5b) is proposed to serve as a mediator of the sex-dependent effects that GH has on liver gene expression [40]. Many genes that were dysregulated in *Pparg*^Δ/Δ^ mice, including *Cux2*, *Acot3* and *Hsd3b5*, were identified as STAT5 targets in the liver [41]. Cux2 is particularly interesting as, as a female-specific transcription factor, it mediates the female-specific expression of a large subset of genes [17]. This complex interaction between sex hormones, GH signaling and liver steatosis may converge on *Cux2* contributing to the perturbation of sex-biased gene expression, such as that of *Fmo3* and *Acot3*, in all four mice models of NAFLD tested herein. However, we cannot exclude an additional direct effect of diabetes, which is a metabolic perturbation shared by the mice models we used. Along this line, Oshida et al. [42] reported that diabetes and obesity in mice inhibit STAT5b activity in male mice, a process the authors named “feminization”. In contrast, the fact that an important number of non-sex-dimorphic genes in CTL become sex-biased in *Pparg*^Δ/Δ^ mice remains difficult to interpret.

One more important point revealed by the microarray analysis concerns the important set of genes that are female-biased in control but not in *Pparg*^Δ/Δ^ mice, which regroups many genes involved in drug metabolism, such as *Fmo3*. Sex dimorphism in drug metabolism-related genes is now well known [9]. The consequence of its perturbation upon liver steatosis is, however, not yet appreciated and would deserve particular attention. Further in line with this idea, a recent study identified hepatocyte PPARα as a relevant sexually dimorphic target in NAFLD, with potential consequences on therapeutic responses targeting this nuclear receptor [43].

Altogether, our results emphasize two major points. Firstly, sex dimorphism of NAFLD in *Pparg*^Δ/Δ^ mice suggests a cross-regulation between PPARγ and sex hormones, whose molecular details still need to be elucidated. Secondly, the important sex-dimorphic expression of genes in the liver is altered upon hepatosteatosis, affecting in particular, but not exclusively, lipid metabolism and drug metabolism pathways. These observations further reinforce the importance of considering the behavior of both sexes in fundamental studies as well as in clinical studies, hopefully leading to more specific and appropriate treatments for men and women.

## 4. Materials and Methods

### 4.1. Animals

All animal experiments and procedures were approved by the Swiss Veterinary Office (VD-1453.4, VD-2560 and VD-2887). Whole-body *Pparg*-null mice (hereafter called *Pparg*^Δ/Δ^) were obtained on a mixed background (Sv129/C56BL/6), as previously described [14]. Fat-specific PPARγ-null mice (*Adipoq-Cre*^tg/+^;*Pparg*^fl/fl^, hereafter called *Pparg*F^Δ/Δ^) were generated as previously described [15]. *ob⁄ob* mice were purchased from Jackson Laboratory (Bar Harbor, ME, USA). *Adipoq-Cre^tg^*^/+;^*Pparg^fl/fl^* and *ob/ob* mice are on a pure C57BL⁄6J genetic background. All animals were kept in a 12:12h light:dark cycle and fed a standard chow diet (cat. 3436, Kliba Nafag, Kaiseraugst, Switzerland) with water ad libitum. All the mice were sacrificed by CO_2_ inhalation between ZT2 and ZT4. Random blocking was used in all experiments. Gonadectomy was performed on mice between 4 and 6 weeks of age, and mice were sacrificed at 20 weeks of age. For more details, see Supplementary Materials and Methods.

### 4.2. Plasma Biochemistry

Plasmatic steroid hormones were measured by LC-MS High Resolution (Q-Exactive, ThermoFisher Scientific, Reinach, Switzerland) as described by Bruce et al. (2014) [44,45]. More details are given in Supplementary Materials and Methods.

### 4.3. Histology and Immunohistochemistry

For all histological analyses, liver left lobes were fixed for 8 h at 4 °C in 4% paraformaldehyde and embedded in paraffin. Paraffin sections (4 μm thickness) were dewaxed and rehydrated before staining with hematoxylin and eosin (H&E) for general histological analysis.

### 4.4. Gene Expression Analysis

Total RNA from the liver was extracted with TRIzol (Invitrogen, ThermoFisher Scientific, Reinach, Switzerland) followed by purification using MagMAX-96 for Microarrays KIT (Ambion, AM1839, ThermoFisher Scientific, Reinach, Switzerland). For the microarray study, RNA was analyzed on Mouse Gene 1.0ST arrays, according to the manufacturer’s instructions (Affymetrix, Santa Clara, CA, USA). Statistical analysis was performed with the statistical language R and various Bioconductor packages (http://www.Bioconductor.org, accessed on 20 April 2021). Normalized expression signals were calculated from Affymetrix CEL files using RMA normalization method. Microarray data were deposited in GEO, series GSE176226. For targeted gene expression analysis, 1 μg of RNA was subjected to reverse transcription using iScript cDNA Synthesis Kit (Bio-Rad Laboratories AG, Cressier, Switzerland). Real-time PCR was performed with SYBR Green (Roche, Basel, Switzerland) using a Fast Real-Time PCR System machine (Applied Biosystem, 7900HT, ThermoFisher Scientific, Reinach, Switzerland). Relative gene expression was determined using the software qBASE (v1.3.5, Biogazelle, Zwijnaarde, Belgium). Results were normalized using 36B4 as housekeeping gene. For primer sequences, see Supplementary Table S1.

### 4.5. Hepatic Lipid Content

For cholesterol and cholesterol esters, frozen liver pieces (10–20 mg) were homogenized in chloroform, isopropanol and Triton X-100 solution (7:11:0.1 *v*/*v*/*v*). Homogenates were centrifuged at 13,200 rpm for 10 min at 4 °C. Supernatants were dried at 50 °C under the hood (O/N). Measurements of hepatic cholesterol and cholesterol esters were performed using a commercial kit (Calbiochem 428901, Merck KGaA, Darmstadt, Germany).

### 4.6. Lipidomics Analysis

Mouse liver tissue (50–100 mg) was homogenized in 10 mL of methyl tert-butyl ether (MTBE) and 3 mL of methanol. Each sample was spiked with 8 nmol of FA 15:0 as internal standard immediately. Then, lipids were extracted according to Matyash et al. [46]. The total amount of FFA was calculated by summing up the quantitative amounts of each lipid species as determined by LS-MS/MS. Values are represented as nmol/g. The total omega-6 and omega-3 FFAs were calculated by summing up the quantitative amounts of all detected omega-6 FFAs (18:2n6, 18:3n6, 20:3n6, 20:4n6 and 22:4n6) and omega-3 FFAs (18:3n3, 20:5n3, 22:5n3 and 22:6n3), respectively. Twenty-week-old mice were assessed, n = 3–5. Detailed protocols are given in Supplementary Materials. For triglyceride species and other lipid classes, 4.5 µL lipid extract was resuspended in 90µl IPA:CHCl3:MeOH (90:5:5 *v*/*v*/*v*), and LM 6000 TG mix (180 pmol for each TG species) was added as internal standard. Data acquisition was performed in data-dependent acquisition mode by an LTQ Orbitrap Velos Pro instrument (ThermoFisher Scientific, Reinach, Switzerland) coupled to a UHPLC (ThermoFisher Scientific, Reinach, Switzerland) according to Fauland et al. [47] at 100.000 mass resolution. Data analysis was done by Lipid Data Analyzer, a custom-developed software tool described in more detail by Hartler et al. [48], with lipid species annotation according to the LipidMAPS shorthand nomenclature [49]. The total amount was calculated by summing up the quantitative amounts of each lipid species as determined by LS-MS/MS; values are represented as nmol/g. Twenty-week-old mice were assessed.

### 4.7. Hepatic Eicosanoids

This analysis was performed as previously described [50]. For more details, see the Supplementary Materials and Methods. Twenty-week-old mice were assessed.

### 4.8. Statistical Analysis

Values, expressed as mean ± SEM, were analyzed using Prism 5.0 (GraphPad Software, San Diego, CA, USA). Unless mentioned, two-way ANOVA and Bonferroni post-test for multiple group comparisons were used to assess statistical significance. *p* values: * <0.05, ** <0.01, *** <0.001 and **** <0.0001.

## Figures and Tables

**Table 1:** Gene ontology analysis of hepatic sex-dimorphic genes in CTL and *Pparg*^Δ/Δ^ mice at 20 weeks.

Group	Gene GO Term	No. of Genes	*p* Value
I (208 genes) Female-biased genes in CTL mice	Microsome	16	2.24 × 10^−9^
	Drug Metabolism	11	1.91 × 10^−8^
	Cytochrome P450	9	2.58 × 10^−6^
	Arachidonic acid metabolism	7	5.16 × 10^−4^
	Endoplasmic reticulum	19	6.23 × 10^−4^
	Linoleic acid metabolism	4	0.01
II (145 genes) Male-biased genes in CTL mice	ncRNA metabolic process	7	3.22 × 10^−3^
	Tubulin	4	3.1 × 10^−4^
	Steroid dehydrogenase activity	4	6.18 × 10^−4^
	Metal ion binding	38	0.04
	Nucleolus	8	5.78 × 10^−3^
III (292 genes) Female-biased genes in γ^Δ/Δ^ mice	Cell surface	24	1.02 × 10^−9^
	Immune response	11	4.06 × 10^−9^
	Immunoglobulin domain	28	4.59 × 10^−9^
	Defense response	26	1.37 × 10^−8^
	Neutrophil-mediated immunity	3	4.43 × 10^−3^
	Fatty acid biosynthesis	5	5.2 × 10^−3^
IV (115 genes) Male-biased genes in in γ^Δ/Δ^ mice	Cell fraction	10	9.87 × 10^−3^
	Cell cycle process	8	5.46 × 10^−3^
	Cellular response to stress	9	1.54 × 10^−3^
	Oxidation reduction	10	0.01
	Mitochondrion	10	0.02

## Data Availability

Microarray data were deposited in GEO and are accessible at the following link: https://www.ncbi.nlm.nih.gov/geo/query/acc.cgi?acc=GSE176226 (from 13 September 2021).

## Supplementary Materials

Supplementary methods, figures and tables are available online at https://www.mdpi.com/article/10.3390/ijms22189969/s1.
