# Supplementary material for: Sex Dimorphism of Nonalcoholic Fatty Liver Disease (NAFLD) in Pparg-Null Mice
Source: Int J Mol Sci. 2021 Sep 15;22(18):9969. doi: 10.3390/ijms22189969 (PMC8467431; doi:10.3390/ijms22189969)
Supplement: Supplementary file 1 [file ijms-22-09969-s001.zip › ijms-1326111.pdf]

International Journal of
Molecular Sciences

MDPI

Supplementary Materials

Sex Dimorphism of Nonalcoholic Fatty Liver Disease (NAFLD) in *Pparg*-Null Mice

Mariano Schiffrin [1], Carine Winkler [1], Laure Quignodon [1], Aurélien Naldi [1], Martin Trötzmüller [2], Harald Köfeler [2], Hugues Henry [3], Paolo Parini [4], Béatrice Desvergne [1] and Federica Gilardi [1,5,*]

[1] Center of Integrative Genomics, Genopode, Lausanne Faculty of Biology and Medicine CH-1015 Lausanne, Switzerland; mariano.schiffrin@gmail.com (M.S.); carine.winkler@unil.ch (C.W.); laurelausanne@gmail.com (L.Q.); aurelien.naldi@gmail.com (A.N.); Beatrice.Desvergne@unil.ch (B.D.)
[2] Core Facility Mass Spectrometry, Medical University of Graz, 8036 Graz, Austria; martin.troetzmueller@medunigraz.at (M.T.); harald.koefeler@klinikum-graz.at (H.K.)
[3] Centre hospitalier universitaire Vaudois (CHUV), Lausanne Faculty of Biology and Medicine, 1011 Lausanne, Switzerland; Hugues.Henry@chuv.ch
[4] CardioMetabolic Unit, Department of Medicine and Department of Laboratory Medicine, Karolinska Insitiutet and Theme Inflammation and Ageing Karolinska University Hospital Huddinge, 14186 Stockholm, Sweden; paolo.parini@ki.se
[5] Faculty Unit of Toxicology, University Center of Legal Medicine, Faculty of Biology and Medicine, Lausanne University Hospital, CH-1000 Lausanne 25, Switzerland
* Correspondence: Federica.gilardi@chuv.ch; Tel.:+41-22-379-55-78 (Faculty Unit of Toxicology, University Center of Legal Medicine, Faculty of Biology and Medicine, Lausanne University Hospital, CH-1000 Lausanne 25, Switzerland

Supplementary Material and Methods

Gonadectomy

Amoxi-Mepha® (50 mg/mL) and Dafalgan® (1 mg/mL) were provided in the water from the day before the surgery up to one week after. Two hours before the surgery, buprenorphine® was administered subcutaneously (0.1 mg/kg). Mice were anesthetized with an intra-peritoneal injection of Xylazine 10 mg/kg and Ketamine 80 mg/kg, and kept on heating pad (at 37 °C) during the surgery. For the sham-operated mice, the procedure was the same except that the gonads were not taken out.

Plasmatic Steroid Hormones

Plasmatic steroid hormones were measured by LC-MS High Resolution (Q-Exactive, ThermoFisher Scientific, Reinach, Switzerland). Plasma samples were thawed at room temperature, vortex-mixed, centrifuged at 3000 g for 5 min. An aliquot of 50µl was spiked with 10 µL of internal standards. After diluting the samples with 5 % (w/v) phosphoric acid, the tubes were centrifuged at 3000 g for 5 min. The analytes were purified using a solid phase extraction (Oasis MCX 96-well plate, Waters). The washing steps were 5% (w/v) NH4OH and 20% (v/v) methanol. The separative column was an Acquity UPLC HSS T3 column (1.8 µm, 1.0 × 100 mm, Waters), the mobile phase comprises of H_2O and 0.01 % formic acid in methanol [1]. The liquid chromatography system was coupled to a Q-Exactive orbitrap mass spectrometer. The calibrants were made by spiking charcoal-dextran stripped serum with prepared steroid solutions from certified standards (Cerilliant-Sigma-Aldrich – Buchs Switzerland and Lipomed – Arlesheim Switzerland).

Histology and Immunohistochemistry

For all histological analysis, left lateral lobes (the biggest lobe) were taken. Liver pieces were fixed during 8 h at 4 °C in 4% paraformaldehyde and embedded in paraffin. All the sections were cut at a 5 µm thickness. Sections were dewaxed and rehydrated before staining. Hematoxilin and eosin (H&E) staining was performed for general histolog-

ical analysis and Sirius Red staining for collagen I and III visualization. For F4/80 immunohistochemistry, slides were incubated in 3% H_2O_2 in PBS for the quenching and in citrate buffer (0.01 M) pH6.0 for antigen retrieval. After 30 min of blocking in 2.5% normal goat serum (NGS) (Dako, Carpinteria, CA), the slides were incubated O/N at 4 °C with the primary antibody (1/800; Abcam, ab6640) and then incubated 40 minutes at room temperature with the secondary antibody (HRP, 1/100; Invitrogen, 629520). After the staining using the enzyme substrate DAB (DAKO), slides were counterstained in Mayer's Hematoxilin, washed, dehydrated and embedded. For LYB-6B.2 neutrophils immunohistochemistry staining, sections were washed in citrate buffer (0.01 M) pH 6.0 for 2 min at RT. For the antigen retrieval, slides were incubated in citrate buffer for 40 min at 95 °C and, after cooling down; they were incubated in 3% H_2O_2 in PBS. The blocking was made in 10% NGS for 30 min at RT. The slides were incubated O/N at 4 °C with the antibody against LYB-6B.2 (1/300; AbD Serotec, MCA771GA) diluted in 2.5% NGS/PBS and then with the secondary antibody anti rat biotinylated (1/200; Vector Laboratories, BA-9400) diluted in 2.5% NGS/PBS. The staining was made with the ABC kit and DAB kit (Vector Laboratories) following the manufacturer instructions. The slides were counterstained in Mayer's Hematoxilin, washed, dehydrated and embedded.

Free fatty acid species

Mouse liver tissue (50–100 mg) were homogenized in 10 ml of methyl-tert-butylether (MTBE) and 3 ml of methanol. Each sample was spiked with 8 nmol of FA 15:0 as internal standard immediately. Then, lipids were extracted according to Matyash et al.[2]. 200 out of 1000 μL lipid extract were dried and dissolved in 50 μL of a pentafluoro benzyl bromide solution (3.4 % in acetonitrile) and 10 μl of N, N-diisopropyl ethanolamine. Samples were resuspended in 50 μl of hexane after evaporation under a stream of nitrogen.

A Trace-DSQ GC-MS (Thermo Scientific, Austin, TX) equipped with a TR-FAME 30 m column was used in splitless mode. The mass spectrometer was run in negative ion chemical ionization (NICI) mode (more details can be given on request). Fatty acids were detected in full scan as carboxylates after loss of the pentafluoro benzyl moiety. Quantitation of fatty acids was performed by correlating integrated areas of fatty acids versus the integrated area of FA 15:0. The total amount of FFA was calculated by summing up the quantitative amounts of each lipid species as determined by LS-MS/MS. Values are represented as nmol/g. The total omega 6 and omega 3 FFAs were calculated by summing up the quantitative amounts of all detected omega 6 FFA (18:2n6, 18:3n6, 20:3n6, 20:4n6 and 22:4n6) and omega 3 FFA (18:3n3, 20:5n3, 22:5n3 and 22:6n3), respectively. 20 weeks mice, n = 3–5.

Hepatic Eicosanoids

All tissues were snap-frozen with liquid nitrogen immediately after collection and stored at −80 °C until extraction. For extraction, each frozen tissue was crushed with a FastPrep ®-24 Instrument (MP Biomedical) in 500 μL of HBSS (Invitrogen, ThermoFisher Scientific, Reinach, Switzerland). After 2 crush cycles (6.5 m/s, 30 s), 10 μL were withdrawn for protein quantification. 300 μL of cold methanol and 5 μL of internal standard (Deuterium labeled compounds) were added to homogenates. After centrifugation at 2000 g for 15 min at 4 °C, supernatants were transferred into 2 mL 96-well deep plates and diluted in H_2O to 2 mL. Samples were then submitted to solid phase extraction (SPE) using OASIS HLB 96-well plate (30 mg/well, Waters) pretreated with MeOH (1 mL) and equilibrated with 10% MeOH (1 mL). After sample application, extraction plate was washed with 10% MeOH (1 mL). After drying under aspiration, lipids mediators were eluted with 1 mL of MeOH. Prior to LC-MS/MS analysis, samples were evaporated under nitrogen gas and reconstituted in 10 μL on MeOH.

LC-MS/MS analyses of eicosanoids were performed as described [3]. Briefly, lipid mediators were separated on a ZorBAX SB-C18 column (2.1 mm, 50 mm, 1.8 μm) (Agilent

Technologies) using Agilent 1290 Infinity HPLC system (Technologies) coupled to an ESI-triple quadruple G6460 mass spectrometer (Agilent Technologies). Data were acquired in Multiple Reaction Monitoring (MRM) mode with optimized conditions (ion optics and collision energy). Peak detection, integration and quantitative analysis were done using Mass Hunter Quantitative analysis software (Agilent Technologies) based on calibration lines built with commercially available eicosanoids standards (Cayman Chemicals, Merck Darmstadt, Germany).

Supplementary Figures

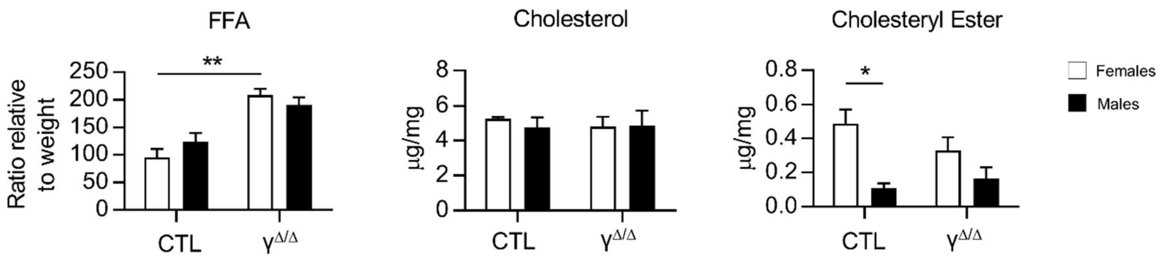

Figure S1. Hepatic lipid profile in *Pparg*$^{\Delta/\Delta}$ mice. Hepatic total FFAs, cholesterol and cholesteryl ester were measured in livers from 20 weeks old mice. N = 3–7. White bars are female and white bars are male data. All data were statistically treated by two way Anova and Bonferroni multiple comparisons. P values: *<0.05 and **<0.01.

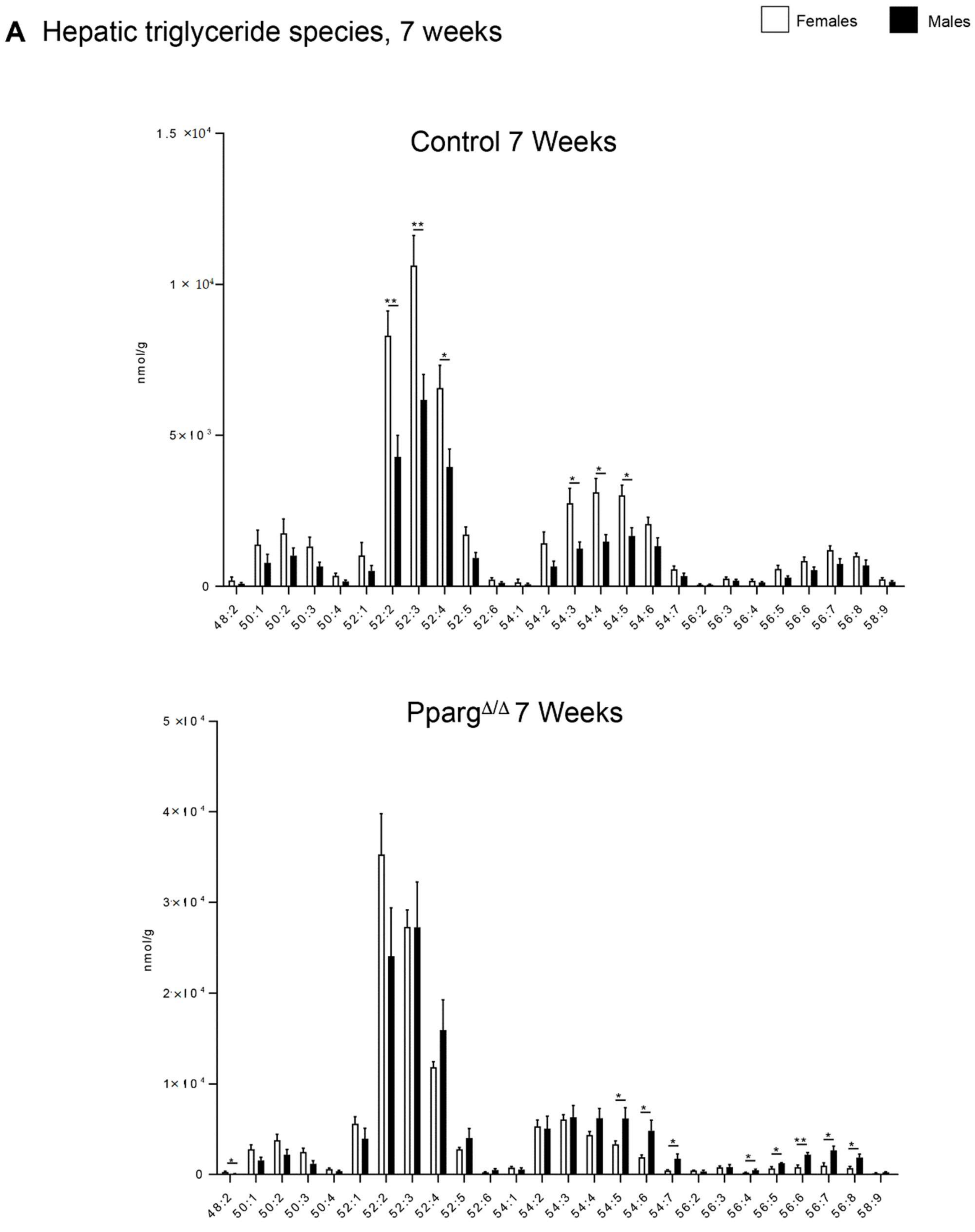
A Hepatic triglyceride species, 7 weeks
Females
Males
Control 7 Weeks
nmol/g
1.5×10^4
1×10^4
5×10^3
0
48:2
50:1
50:2
50:3
50:4
52:1
52:2
52:3
52:4
52:5
52:6
54:1
54:2
54:3
54:4
54:5
54:6
54:7
56:2
56:3
56:4
56:5
56:6
56:7
56:8
58:9
Pparg$^{\Delta/\Delta}$ 7 Weeks
nmol/g
5×10^4
4×10^4
3×10^4
2×10^4
1×10^4
0

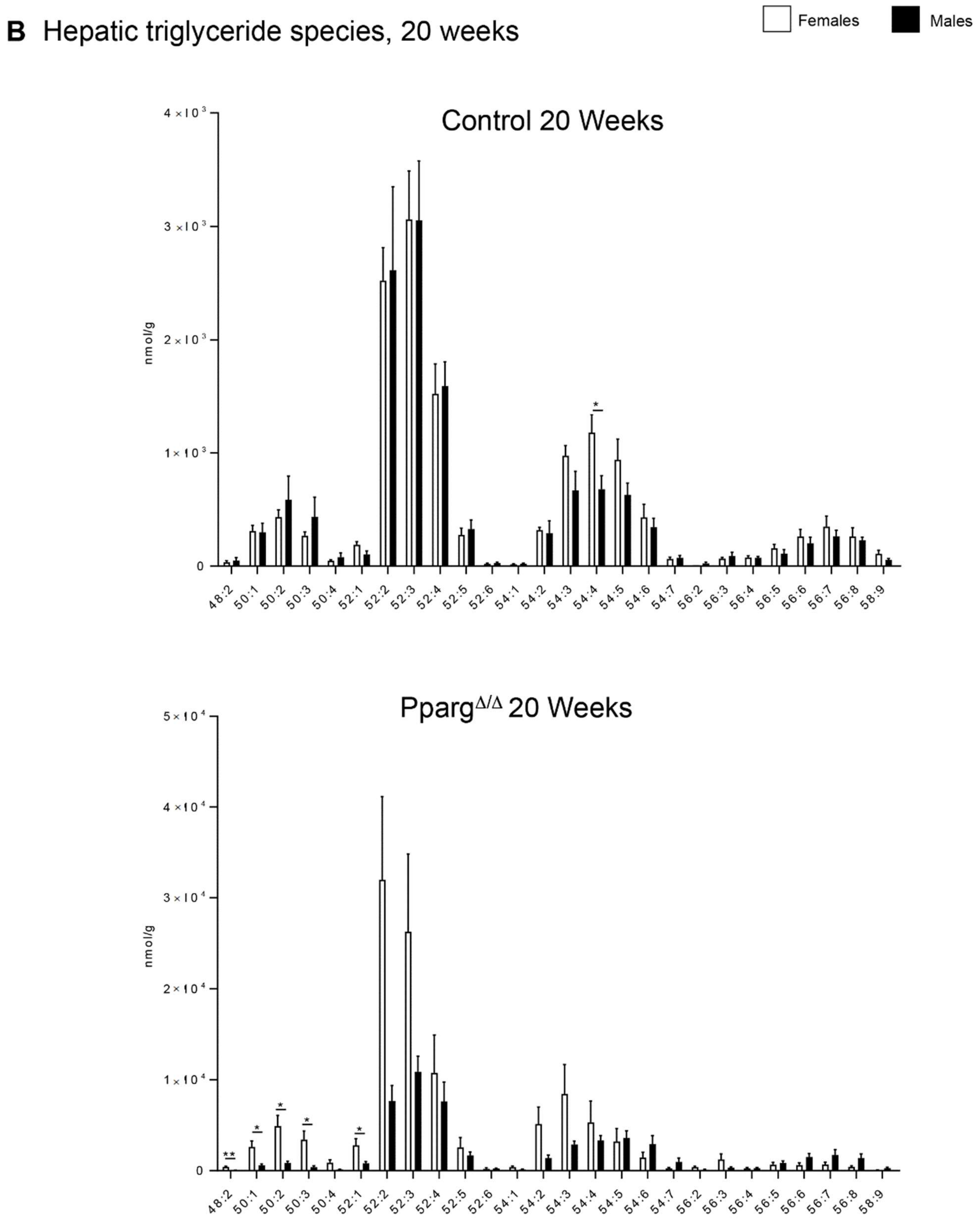
B Hepatic triglyceride species, 20 weeks
Females
Males
Control 20 Weeks
nmol/g
Pparg$^{\Delta/\Delta}$ 20 Weeks
48:2
50:1
50:2
50:3
50:4
52:1
52:2
52:3
52:4
52:5
52:6
54:1
54:2
54:3
54:4
54:5
54:6
54:7
56:2
56:3
56:4
56:5
56:6
56:7
56:8
58:9

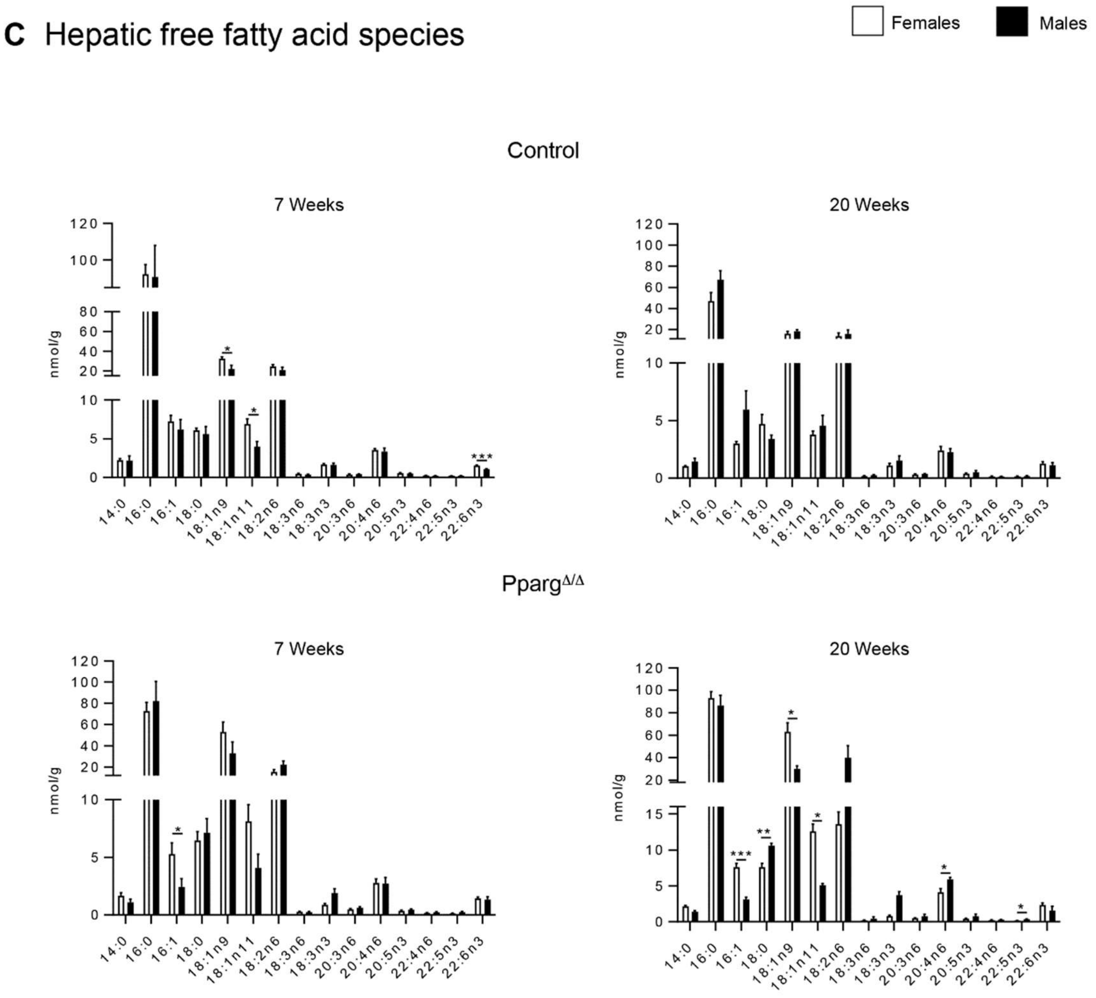

Figure S2. Absolute hepatic concentrations of TG and FFA species measured at 7 and 20 weeks. Hepatic triglyceride species in control and *Pparg*$^{\Delta/\Delta}$ mice at 7 **(A)** and 20 weeks **(B),** in females (white bars) and males (black bars). n = 5. (**C**) Hepatic FFA species in control and *Pparg*$^{\Delta/\Delta}$ mice at 7 and 20 weeks. n = 3–5. P values; *<0.05, **<0.01, ***<0.001 and ****<0.0001. P values (*<0.05, **<0.01, ***<0.001 and ****<0.0001) were calculated by student T.test between males and females in *Pparg*$^{\Delta/\Delta}$.

Supplementary Figure S3A&B

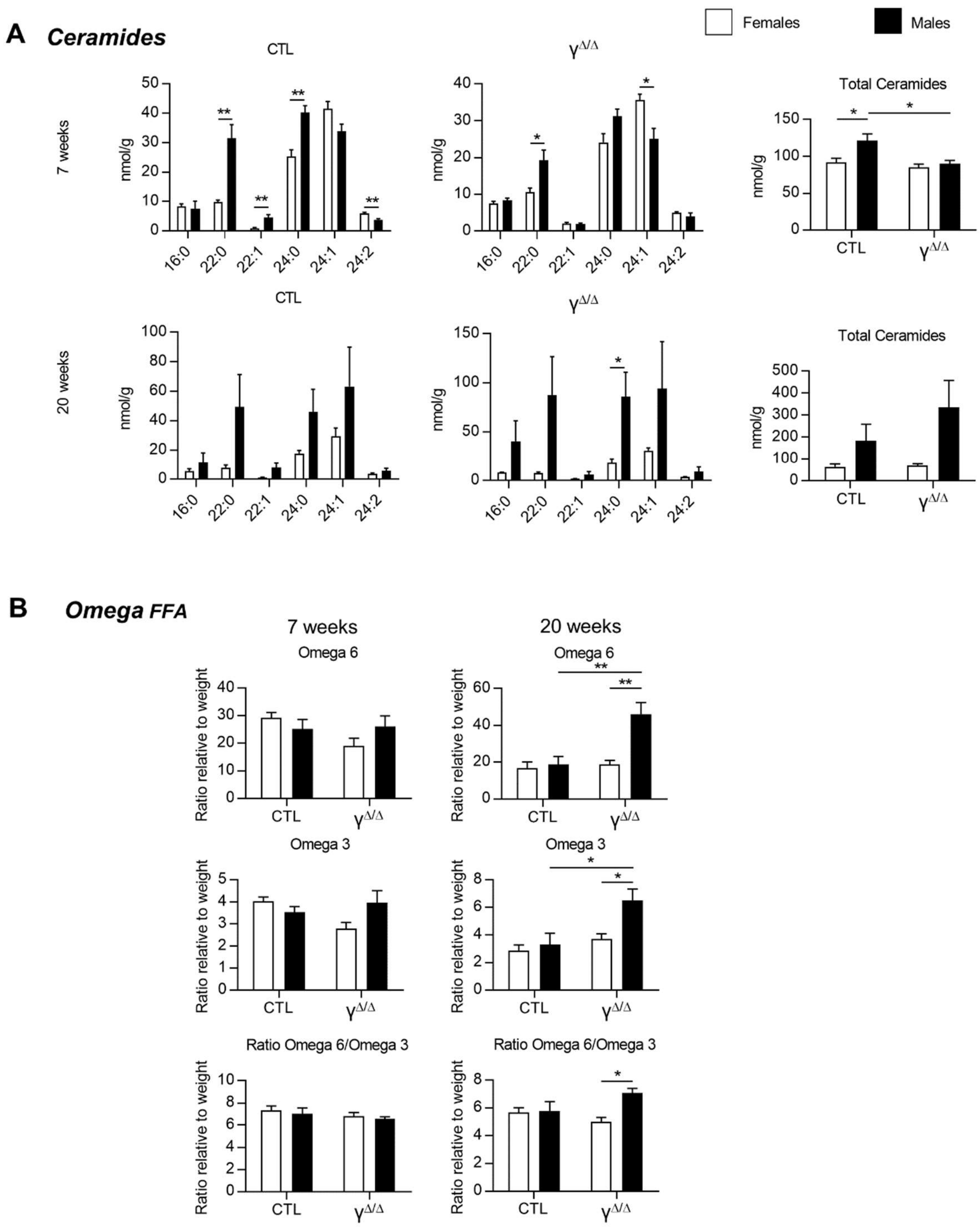

Supplementary Figure S3C

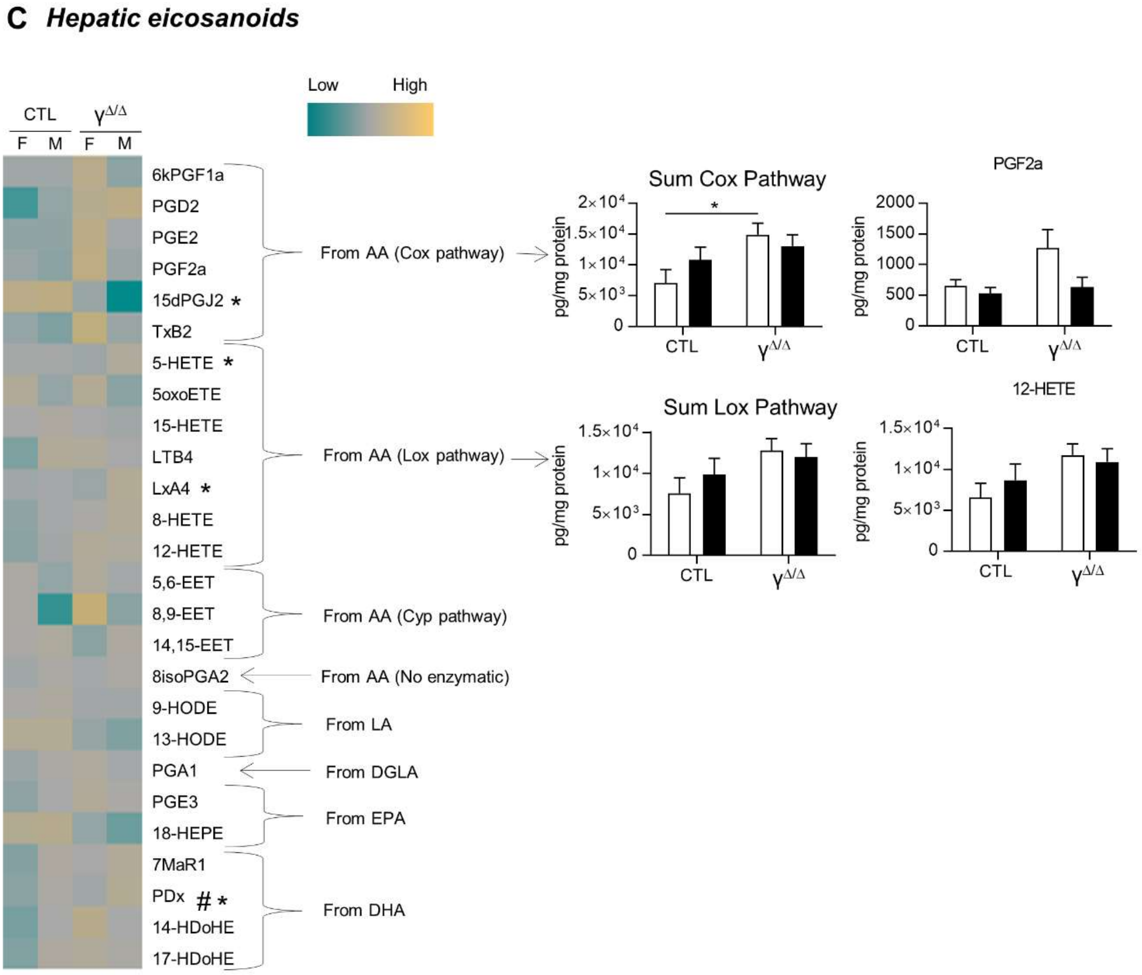

Figure S3. Hepatic ceramides and eicosanoids in *Pparg*$^{\Delta/\Delta}$ mice at 20 weeks. **(A)** Differences in ceramides (left and middle panels), as calculated by student t-test. Total hepatic ceramides (right panels) measured as the sum of the different ceramide species; n = 4–6. **(B)** Hepatic Omega-6 represents the sum of 18:2n6, 18:3n6, 20:3n6; 20:4n6 and 22:4n6. Omega-3 represents the sum of 18:3n3, 20:5n3, 22:5n3 and 22:6n3. N = 3–6. # and * indicate statistically significant difference ($P < 0.05$) between females and males in control and *Pparg*$^{\Delta/\Delta}$ mice, respectively (student t.test). White bars are female and black bars are male data. **(C)** Heatmap showing hepatic content of arachidonic acid (AA), linoleic acid (LA), dihomo-γ-linolenic acid (DGLA), eicosapentaenoic acid (EPA) and docosahexaenoic acid (DHA) derivatives. Colors encoding the rectangular cells represent the mean of the four groups for each lipid species; absolute values were centered to 1 and changed in log2

(log(V/mean;2)). Yellow and green correspond to higher and lower relative amounts, respectively. "Cyp pathway" represent the eicosanoids synthetized through the cytochrome P450 pathway. # and * indicate statistically significant difference (P < 0.05) between females and males in control and *Pparg*$^{\Delta/\Delta}$ mice, respectively (student t.test). **On the right** Hepatic eicosanoid contents showed as absolute values (pg/mg protein). AA (Cox) and AA (Lox) represent the sum of the eicosanoids synthesized through the cyclooxygenase pathway and the lipoxygenase pathway, respectively. Absolute levels of the most abundant eicosanoid from each pathway such as Prostaglandin F2alpha (PGF2a) for the Cox pathway and 12-Hydroxyeicosatetraenoic acid (12-HETE) for the Lox pathway. White bars are female and black bars are male data. P values (* < 0.05) were calculated by student T.test between males and females in *Pparg*$^{\Delta/\Delta}$.

Supplementary Figure S4

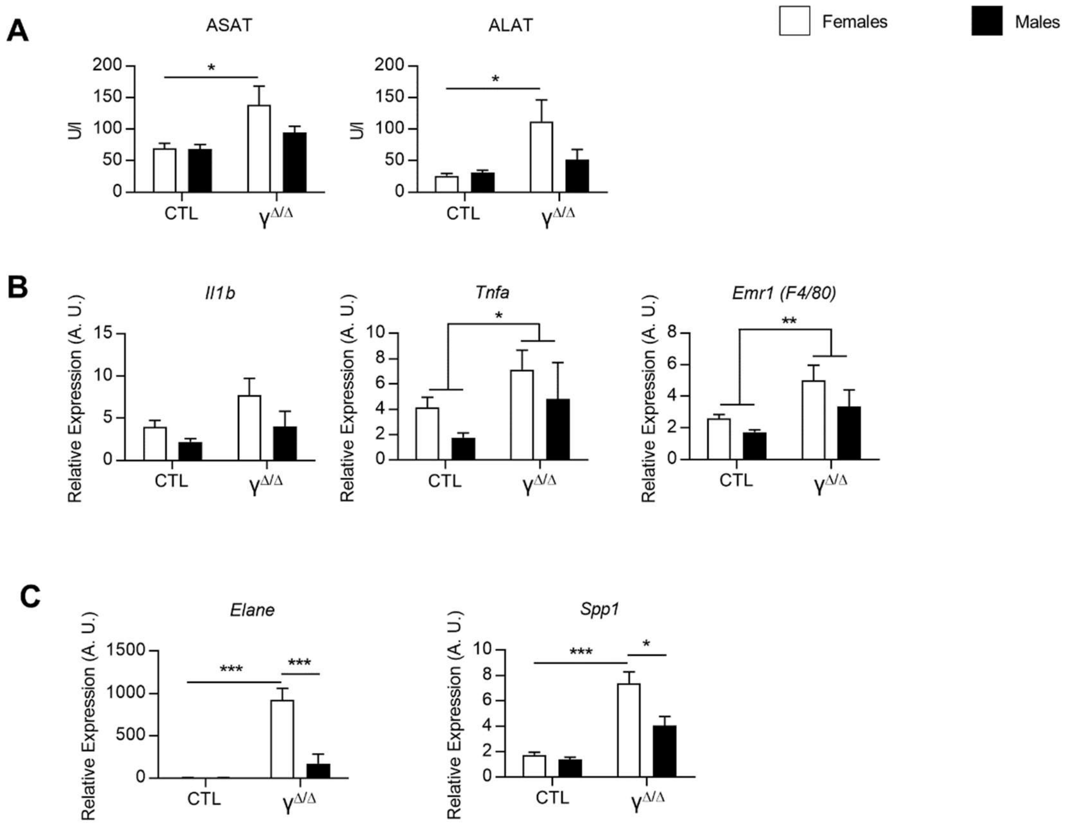

Figure S4. Markers of liver damages in *Pparg*$^{\Delta/\Delta}$ mice**. (A)** Plasmatic measurement of ALAT (alanine aminotransferase) and ASAT (aspartate aminotransferase) at 20 weeks. N = 3–6. **(B)** Gene expression of inflammatory markers in the liver at 20 weeks. **(C)** Gene expression of neutrophil markers Elastase Neutrophil Expressed (Elane) and osteopontin (spp1) at 20 weeks. N = 3–9. White bars are female and black bars are male data. P values (*<0.05, **<0.01, and ***<0.001) were calculated by two-way Anova and Bonferroni multiple comparisons.

Supplementary Figure S5

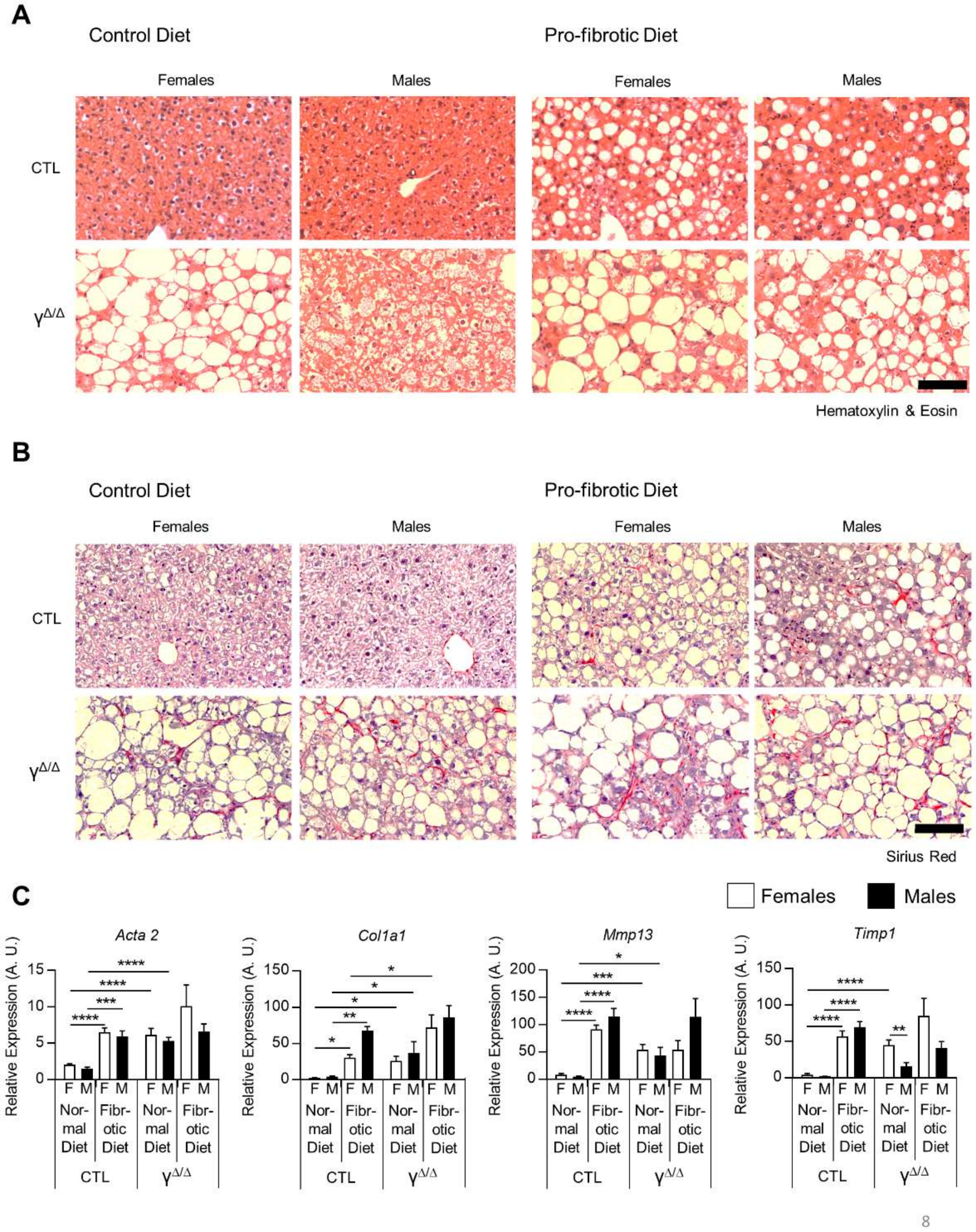

Figure S5. No sex dimorphism observed in hepatic fibrosis with the Pro-fibrotic treatment in *Pparg*$^{\Delta/\Delta}$ mice. Mice were fed with control diet or the pro-fibrotic diet choline-deficient and methionine-reduced to 0.1% with 12%kcal fat for 6 weeks, starting at 20 weeks. H&E **(A)** and Sirius Red staining **(B)** of liver sections in male and female mice, as indicated. Scale bars correspond to 100µm. **(C)** Gene expression of fibrotic markers measured by RT-qPCR. Alpha-actin-2 (*Acta2*), collagen 1a1 (*Col1a1*), matrix metalloproteinase 13 (*Mmp13*), tissue inhibitor of metalloproteinase 1 (*Timp1*), n = 5–9. White bars are female and black bars are male data. P values (* <0.05, **<0.01, ***<0.001 and ****<0.0001) were calculated by two-way Anova and Bonferroni multiple comparisons.

Supplementary Figure S6

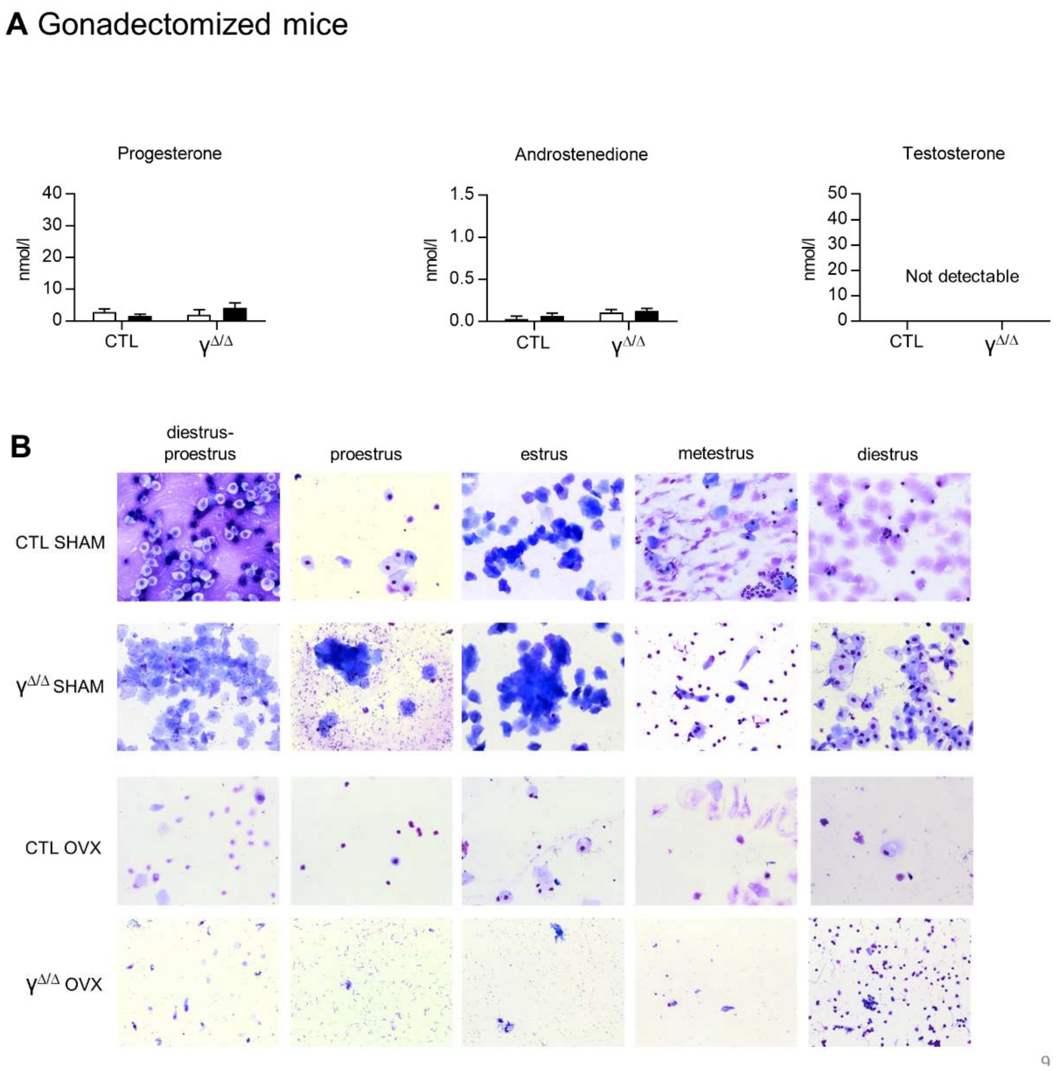

Figure S6. Gonadectomized mice have low or no detectable sex hormones and female mice do not cycle. **(A)** Plasmatic steroid hormones measured in gonadectomized mice at 20 weeks, n = 4–9. **(B)** Vaginal smears were performed each day during 5 days followed by May Gruenwald Gemsa staining. The different stages of the estrous cycle are determined by the type of cells present in the vaginal smears. Proestrus is characterized by the presence of largely small, round, nucleated epithelial cells with none or few leucocytes. At proestrus, mostly nucleated and few cornified cells are present along with some leukocytes. In estrus, only cornified cells are present. Metestrus is characterized by cornified epithelial cells and leukocytes while at diestrus, which is the longest phase, only leukocytes are seen accompanied by few epithelial nucleated cells [4]. Cornified cells are well recognizable during the estrus in SHAM mice, both in control and *Pparg*$^{\Delta/\Delta}$ mice. White bars are female and black bars are male data.

Table S1. sequences of the primers used for qPCR.

Genes	Fwd	Rev
36b4	CCGATCTGCAGACACACACT	ACCCTGAAGTGCTCGACATC
Acacb	AGTCTTCCGTGCCTTTGTAC	TTCTGCAAACTCATCCCTCG
Acot3	GCTGTGACCTACCTGCTCAGTCA	ATATAGAGCCATTGATGATGACAGCGG
Acta2	GTGAAGAGGAAGACAGCACAG	GCCCATTCCAACCATTACTCC
Aldoc	CTGGAGAGGACAAAGGGATAATG	CATGGTTGGCGATGTAGAGG
Alox5	TGTCTGAGGTGTTTGGTATCG	AAGGCCATACTCGCAGATAAG
Alox5ap	CTTTGAGCGGGTCTACACTG	ACAAACAGGTACATCAGTCCG

Col1a1	CATAAAGGGTCATCGTGGCT	TTGAGTCCGTCTTTGCCAG
Crat	ACCTCTTACGCCATTGCTATG	TGACACGGAGAAGTTGATATGG
Cux2	TCAGTCAACAGCTCCATTCG	CGTCCTTCAACAGCCTCAG
Cyp17a1	AGAGTTTGCCATCCCGAAG	AACTGGGTGTGGGTGTAATG
Elane	TACTGGCATTGTTCCTGGG	ACGAAGTTCCTGGCAATGAG
Elovl5	TTTCGATGCGTCACTCAGTAC	GGTCCCAGCCATACAATGAG
Emr1 (F4/80)	ACCACAATACCTACATGCACC	AAGCAGGCGAGGAAAAGATAG
Fads1	GTCTACCTGCTTCACATCCTG	TGCCCAAAGTCATGCTGTAG
Fads2	GCTCATCCCTATGTACTTCCAG	CTCCCAAGATGCCGTAGAAAG
Fmo3	CACCATCCCCATAACTGACC	CCATACCATTTGAACTTTTCCCC
G0S2	CAGTCCCTCCACAAGCAG	TCTGTGTCTCCCTTCTCTGTC
G6pdx	GCCAACCGTCTATTCTACCTG	CAGTTGATTGGAGCTCTGTAGG
Gpam	ATCGAAGGTCACTACAATGGC	CTTCAAGGAAAATGGCTGTGC
Hilpda	TGCTGGGCATCATGTTGACC	TGACCCCTCGTGATCCAGG
Hsd3b5	AGAGGATTGTCCGAATGTTGG	CATCCAGAATGTCTCCCTTCAG
IL-1b	CGTTCCCATTAGACAACTGC	GCTCATGGAGAATATCACTTGT
Lta4h	TCTACTTAGAGCGCCACATTTG	GTCCTTCAGATCAACCACGAG
Mmp13	TTGATGCCATTACCAGTCTCC	ACATGGTTGGGAAGTTCTGG
Plin2	GGATAAGCTCTATGTCTCGTGG	GTCTGGCATGTAGTCTGGAG
Pnpla2 (ATGL)	ATATCCCACTTTAGCTCCAAGG	CAAGTTGTCTGAAATGCCGC
Scd1	CTGACCTGAAAGCCGAGAAG	AGAAGGTGCTAACGAACAGG
Spp1 (OPN)	TGGCTATAGGATCTGGGTGC	ATTTGCTTTTGCCTGTTTGG
Timp1	CTCAAAGACCTATAGTGCTGGC	CAAAGTGACGGCTCTGGTAG
TNFa	AGGGTCTGGGCCATAGAACT	CCACCACGCTCTTCTGTCTAC

Table S2. Gene Ontology analysis on microarray gene expression data.

Group I: Female-biased genes in CTL but not in *Pparg*$^{\Delta/\Delta}$ mice

Term	Number of genes	P. value	Genes
Microsome	16	2.24×10^{-9}	CYP2C37, NCEH1, CYP2B9, POR, CYP4A10, FMO4, CYP2A22, CYP4A32, KCNJ8, FMO1, FMO2, CYP2B19, CYP4A14, CYP2C38, JPH1, CYP2C39
Drug metabolism	11	1.91×10^{-8}	FMO4, CYP2A22, CYP2C37, FMO1, FMO2, CYP2B9, CYP2B19, GSTT2, CYP2C38, CYP2C39, CYP3A44
Cytochrome P450	9	2.58×10^{-6}	CYP4A10, CYP2A22, CYP2C37, CYP4A32, CYP2B9, CYP2B19, CYP4A14, CYP2C38, CYP2C39, CYP3A44
Arachidonic acid metabolism	7	5.16×10^{-4}	CYP4A10, CYP2C37, CYP4A32, CYP2B9, CYP2B19, CYP4A14, CYP2C38, CYP2C39
Endoplasmic reticulum	19	6.23×10^{-4}	CYP2C37, NCEH1, CYP2B9, UGCG, LASS6, POR, CYP4A10, FMO4, CYP2A22, INPP5K, SULF2, CYP4A32, FMO1, FMO2, CYP2B19, CYP2C38, CYP4A14, JPH1, CYP2C39
Linoleic acid metabolism	4	0.01	CYP2C37, CYP2C38, CYP2C39, CYP3A44

Group II: Male-biased genes in CTL but not in *Pparg*$^{\Delta/\Delta}$ mice			
**Term**	**Number of genes**	**P. value**	**Genes**
NcRNA metabolic process	7	3.22 × 10^{-3}	EIF4A3, CARS, YARS, AARS, BOP1, FTSJ3, MARS
Tubulin	4	3.11 × 10^{-4}	TUBB2B, TUBB2A, TUBB2C, TUBA4A
Steroid dehydrogenase activity	4	6.19 × 10^{-4}	HSD3B2, HSD3B3, HSD3B4, DHRS9
Metal ion binding	38	0.03	APEX2, GCLC, SLC39A10, PNKD, LNX2, NFXL1, SLC11A2, PCGF2, TRIM2, NUDT7, U2AF1, RNF11, PHOSPHO1, BCL6, PHF20L1, DTX4, CARS, TRIP4, MICAL2, SCN2A1, EPHX2, FAM188A, ITPA, B3GAT3, CAPN11, SLC41A2, EIF2S2, AOX1, ARSA, ZMYND19, TNK2, ZFHX2, TRIP6, ACCN5, SCARA5, MAP3K13, UNC13B, NEK6
Nucleolus	8	5.78 × 10^{-3}	SENP3, TSR1, NOP16, YPEL2, BOP1, SRP19, FTSJ3, MARS

Group III: Female-biased genes in *Pparg*$^{\Delta/\Delta}$ but not in CTL mice			
**Term**	**Number of genes**	**P. value**	**Genes**
cell surface	24	1.02 × 10^{-9}	LY75, PTPRC, CD8B1, CD8A, H2-M2, CD3E, H2-AB1, ITGB2, ITGA4, IL7R, MMP14, CD74, BTLA, CD83, TCRG-V3, ITGAX, CLEC12A, LY6G, KLRB1F, CD22, H2-AA, CD79B, CLEC7A, CD79A
Immune response	11	4.00 × 10^{-9}	BTLA, PTPRC, KLHL6, CD3E, LAX1, LCK, UNC93B1, MALT1, CD79B, CD79A, CLEC7A
Immunoglobulin domain	28	4.59 × 10^{-9}	CD8A, FCRL1, CD96, TCRG-V3, UNC5B, GP49A, CD22, EMB, MR1, IGHMAC38.205.12, CD7, CD8B1, CD3G, CD3E, IGK-V28, SLAMF6, MALT1, SLAMF7, SIRPA, CD84, BTLA, CD83, CD300A, LILRB4, CD79B, CD79A, CD226, LOC435333
Defense response	26	1.37 × 10^{-8}	TLR1, CXCL9, ITGB2, CXCL11, CD74, TLR9, PYCARD, IL1B, THBS1, IL1A, NFKBIZ, PTPRC, NGP, CAMP, ELANE, MYO1F, MALT1, CHI3L3, LSP1, CCR2, H2-AA, MPO, CLEC7A, CD14, CTSG, PTAFR
Neutrophil mediated immunity	3	4.43 × 10^{-3}	ELANE, MYO1F, CTSG
Fatty acid biosynthesis	5	5.20 × 10^{-3}	SCD2, TBXAS1, FADS1, FADS2, HPGDS

Group IV; Male-biased genes in *Pparg*$^{\Delta/\Delta}$ but not in CTL mice			
**Term**	**Number of genes**	**P. value**	**Genes**
Cell fraction	10	9.87 × 10^{-3}	CYP2C54, HPRT, INPP5J, PTGES, CES6, CYP21A1, ACMSD, SLC30A5, PNPLA6, ABCB4
Cell cycle process	8	5.46 × 10^{-3}	TXNL4B, ATF6B, MLH1, MDM2, CHEK1, DDIT3, MIS12, JMY
Cellular response to stress	9	1.54 × 10^{-3}	AEN, MLH1, HSPA1A, CHEK1, HSPA5, CRY1, OBFC2A, DDIT3, JMY
Oxidation reduction	10	0.01	HSD17B10, 9130409I23RIK, CYP2C54, CYP2C44, ADH4, CYP21A1, 4933437F05RIK, PNPO, RSAD1, BDH2
Mitochondrion	10	0.02	MRPS27, HSD17B10, AGXT2L1, MRPS18B, RSAD1, FH1, MRPL46, SLC25A15, KEG1, EARS2

Gene Ontology analysis performed with the software DAVID available on web (https://david.ncifcrf.gov/home.jsp).

References

1. Bruce, S.J., Rey, F., Beguin, A., Berthod, C., Werner, D., Henry, H. Discrepancy between radioimmunoassay and high performance liquid chromatography tandem-mass spectrometry for the analysis of androstenedione. *Anal Biochem.* **2014**;*455*:20–25.
2. Matyash, V., Liebisch, G., Kurzchalia, T.V., Shevchenko, A., Schwudke, D. Lipid extraction by methyl-tert-butyl ether for high-throughput lipidomics. *J Lipid Res*. **2008**; *49*, 1137–1146.
3. Le Faouder P., Baillif, V., Spreadbury, I., Motta, J.-P., Rousset, P., Chêne, G., Guigné, C. Tercé, F., Vanner, S. Vergnolle, N., et al LC-MS/MS method for rapid and concomitant quantification of pro-inflammatory and pro-resolving polyunsaturated fatty acid metabolites., *J Chrom B*, **2013**, *932*, 123–133
4. Byers, S.L., Wiles, M.V., Dunn, S.L., Taft, R.A. Mouse estrous cycle identification tool and images. *PLoS One*. **2012**, *7*, e35538.
